# Sodium Manganese Ferrite Water Splitting Cycle: Unravelling the Effect of Solid–Liquid Interfaces in Molten Alkali Carbonates

**DOI:** 10.1021/acsami.4c00549

**Published:** 2024-06-19

**Authors:** Joseba Udaeta, Mikel Oregui Bengoechea, Francesco Torre, Nerea Uranga, Marta Hernaiz, Beatriz Lucio, Pedro Luis Arias, Elena Palomo del Barrio, Stefania Doppiu

**Affiliations:** † Department of Chemical and Environmental Engineering, School of Engineering, University of the Basque Country UPV/EHU, Plaza Ingeniero Torres Quevedo, 1, 48013 Bilbao, Spain; ‡ Centre for Cooperative Research on Alternative Energies (CIC energiGUNE), Basque Research and Technology Alliance (BRTA), Alava Technology Park, Albert Einstein 48, 01510 Vitoria-Gasteiz, Spain; § Tekniker, Basque Research and Technology Alliance (BRTA), Parke Teknologikoa, Iñaki Goenaga, 5 20600 Eibar, Gipuzkoa, Spain; ∥ Ikerbasque, Basque Foundation for Science, Bilbao 348013, Spain

**Keywords:** thermochemical water splitting, sodium manganese ferrite cycle, atomic substitution, carbonation, decarbonation, hydrogen production

## Abstract

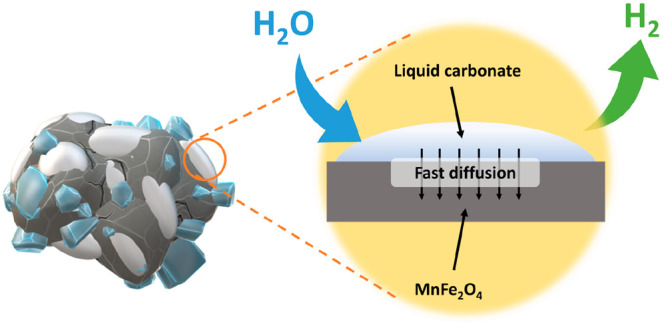

In this work, the Na_2_CO_3_ of the sodium manganese ferrite thermochemical cycle was substituted by different eutectic or eutectoid alkali carbonate mixtures. Substituting Na_2_CO_3_ with the eutectoid (Li_0.07_Na_0.93_)_2_CO_3_ mixture resulted in faster hydrogen production after the first cycle, shifting the hydrogen production maximum toward shorter reaction times. Thermodynamic calculations and *in situ* optical microscopy attributed this fact to the partial melting of the eutectoid carbonate, which helps the diffusion of the ions. Unfortunately, all the mixtures exhibit a significant loss of reversibility in terms of hydrogen production upon cycling. Among them, the nonsubstituted Na mixture exhibits the highest reversibility in terms of hydrogen production followed by the 7%Li-Na mixture, while the 50%Li-Na and Li-K-Na mixtures do not produce any hydrogen after the first cycle. The loss of reversibility is attributed to both the formation of undesired phases and sintering, the latter being more pronounced in the eutectic and eutectoid alkali carbonate mixtures, where the melting of the carbonate is predicted by thermodynamics.

## 1 Introduction

In recent years, a growing number of countries and organizations have undertaken different strategies to reach the aim of net-zero carbon emissions by 2050. In pursuit of this climate target, green hydrogen produced from renewable sources is thought to be a game changer.^1−3^ On the one hand, it can be used to decarbonize hard-to-electrify sectors such as heavy-duty transport and heavy industry, e.g., steel, cement, chemicals and fertilizers production. On the other hand, it is considered a versatile energy storage medium for smart grids to support the integration of intermittent renewable generation.^4,5^ Nevertheless, less than 1% of the hydrogen is currently produced from renewable sources, with the vast majority being produced using fossil fuels.^6−8^ In this context, water-splitting thermochemical cycles are considered as a promising alternative due to their very low global warming potential (1.2 kg CO_2_ kg^–1^ H_2_).^9^ However, this promising technology is still not cost-effective or industrially established.^10^

A water-splitting thermochemical cycle (WSTC) is a cyclic succession of chemical reactions in which thermal energy is used to produce hydrogen and oxygen from water vapor in the presence of a thermochemical material. Thus, the overall reaction of one thermochemical cycle is equivalent to the direct thermal water decomposition since the only substance consumed in the process is water, and the thermochemical material is regenerated after every cycle.^8,11,12^ The main advantages of WSTCs over the direct thermal water decomposition are (a) the reduction of the operating temperature to 500–1800 °C and (b) not requiring downstream hydrogen and oxygen separation since they are released in different reaction steps.^11,13^

Mixed ferrites (MFe_2_O_4_) are promising materials for water splitting. In these oxides the Fe^2+^ of the ferrite is partially substituted by other bivalent cations such as Ni, Co, Zn, and Mn.^14−17^ With these materials, H_2_ yields similar to those of the Fe_3_O_4_/FeO can be achieved but at considerably lower reduction temperatures.^15,16^ Among mixed ferrites, those containing both Ni and Co exhibit the best performance and a superior durability at the temperatures encountered in a “real” CSP plant.^17,18^ Conversely, those ferrites containing Zn and Mn face significant challenges such as zinc volatilization in the case of the former and phase stability in the case of the latter.^17^ However, Mn-ferrites exhibit a major advantage over Zn-ferrites, as they present faster water-splitting kinetics.^19^ In any case, the main limitation of conventional two-step cycles based on mixed ferrites is that the direct reduction of ferrites typically needs temperatures up to 1400 °C.

In this regard, the sodium manganese ferrite thermochemical cycle, proposed by Tamaura,^20^ is a two-step WSTC with a relatively low operating temperature, i.e., 700–800 °C,^21^ and whose feasibility has already been proven in a small solar concentration facility.^22^ Furthermore, the cycle can operate under isothermal conditions, which would result in the elimination of the energy losses due to heating and cooling and the reduction of the stresses related to thermal shocks.^22^ The chemical reactions taking place in this thermochemical cycle are presented below:
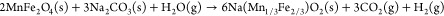
1
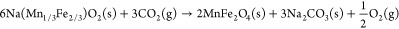
2

In the first step, i.e., the water splitting step (WS), water vapor is reduced into hydrogen due to the oxidation of the Mn^2+^ of the manganese ferrite (MnFe_2_O_4_) when the latter reacts with sodium carbonate (Na_2_CO_3_) to form sodium ferrimanganite (Na(Mn_1/3_Fe_2/3_)O_2_) and carbon dioxide. In the second step, i.e., the reduction step (RE), the Mn^3+^ of the Na(Mn_1/3_Fe_2/3_)O_2_ is reduced under a carbon dioxide atmosphere leading to the regeneration of the thermochemical material, i.e., MnFe_2_O_4_ and Na_2_CO_3_, and the production of oxygen.^23,24^

However, according to the literature, the reaction scheme of the sodium manganese thermochemical cycle is of greater complexity. The WS step (reaction 1) is actually divided into two discrete reaction steps.^25,26^ Initially, a nonoxidative partial decarbonation takes place: the Na_2_CO_3_ partially reacts with the MnFe_2_O_4_ yielding a mixed metal oxide intermediate, MnO*2NaFeO_2_, CO_2_, and the remaining Na_2_CO_3_ (reaction 3). Later, the remaining Na_2_CO_3_ reacts with water vapor and the MnO*2NaFeO_2_ intermediate to form Na(Mn_1/3_Fe_2/3_)O_2_, CO_2_, and H_2_ (reaction 4).
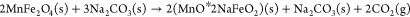
3
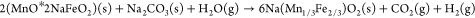
4

Despite having a great potential for practical applications, there are certain aspects that need to be improved in the sodium manganese ferrite thermochemical cycle in order to guarantee its industrial implementation.^27^ The main drawback of this thermochemical cycle is the continuous decrease of the amount of hydrogen produced upon cycling.^25,28,29^ One of the main causes of this reversibility loss is the difficulty to achieve a total regeneration of the initial thermochemical material, MnFe_2_O_4_ and Na_2_CO_3_, from the NaMn_1/3_Fe_2/3_O_2_.^28^ Another cause is related to morphological variations of the material such as sintering and the coalescence and incorporation of the Na_2_CO_3_ over the MnFe_2_O_4_, what leads to the reduction of the interface number sites between Na_2_CO_3_, MnFe_2_O_4_, and water vapor.^29^ As sintering and coalescence are thermally activated processes, decreasing the operating temperature or decreasing the reaction time could be beneficial for the reversibility of the cycle. Moreover, improving the reaction kinetics is highly desired to increase the overall process efficiency.^30^

In this regard, the substitution of the Na_2_CO_3_ by the different eutectic or eutectoid alkali carbonate mixtures that melt at lower temperatures than Na_2_CO_3_ could be an adequate strategy to decrease the operation temperature, improve the reaction kinetics and enhance the reversibility of the cycle. On the one hand, the decomposition temperatures of alkali carbonates are directly related to their melting temperature: the thermal decomposition of pure alkali carbonates is known to take place after finishing its melting process.^31^ Hence, reducing the melting temperature of the alkali carbonates could result in a reduction of the alkali carbonate decomposition temperature. This decrease could have a beneficial effect in the temperature and reaction kinetics of the nonoxidative partial decarbonation reaction (reaction 3) and, thus, on the WS reaction (reaction 4). On the other hand, the addition of an alkali carbonate other than Na_2_CO_3_ to the reaction mixture will have a significant effect on the nature of the reaction intermediates and hence on the reversibility of the cycle.

In this work, we assess the effect of substituting Na_2_CO_3_ with different eutectic or eutectoid alkali carbonate mixtures in the reversibility and WS kinetics of the sodium manganese ferrite thermochemical cycle. For this purpose, a three-step experimental approach was carried out. Initially, the melting and decomposition temperature of the eutectic or eutectoid alkali carbonate mixtures were evaluated in the absence and presence of MnFe_2_O_4_. Later, the effect of alkali carbonate substitution was evaluated under nonoxidative conditions, i.e., in the absence of water, for several decarbonation-carbonation thermochemical cycles. Finally, the effect of alkali carbonate substitution in the production of hydrogen was evaluated under oxidative conditions, i.e., presence of water, for several WS-RE cycles (reaction 1 and reaction 2).

## 2 Material and Methods

### 2.1 Selection of Carbonate Compositions and Thermodynamic Calculations

The different mixed alkali carbonates were selected based on equilibrium phase diagrams. The binary Na_2_CO_3_-L_i2_CO_3_ phase diagram was constructed using the FactSage program,^32^ which is based on the CALPHAD (calculation of phase diagram) methodology. The FactPS and FTsalt databases were used. The Na_2_CO_3_-L_i2_CO_3_ diagram is reported in Figure S1. Two different compositions were chosen: (i) the eutectic (Na_0.48_Li_0.52_)_2_CO_3_ and (ii) the eutectoid (Na_0.93_Li_0.07_)_2_CO_3_. The first one has a congruent melting point of 499 °C, while the second one starts melting at 631 °C. In addition, a ternary eutectic (Na_0.33_Li_0.32_K_0.34_)_2_CO_3_ mixture was chosen. This composition has been previously investigated as a molten salt for thermal energy storage (TES) and heat transfer fluid (HTF) and melts at around 400 °C.^33,34^ The FactSage software was used to perform equilibrium calculations of the MnFe_2_O_4_-carbonate mixtures at different temperatures. Liquid, solid, and gaseous compounds were considered in the calculations, using the FactPS, FTsalt and FToxid databases. Calculations both under an inert atmosphere (Ar) and CO_2_ were performed. A big volume of gas (1000 mol of Ar or CO_2_) was included in the starting reactants. In this way, any possible gaseous products coming from the MnFe_2_O_4_-carbonate decomposition would be highly diluted in the starting Ar or CO_2_, leading to a neglectable effect on the thermodynamic equilibrium. This is necessary to be as close as possible to the open configuration of the thermobalances used to experimentally test the mixtures (details in Section 2), where the gaseous products are removed continuously.

### 2.2 Preparation of the Mixtures

**Figure 1:**
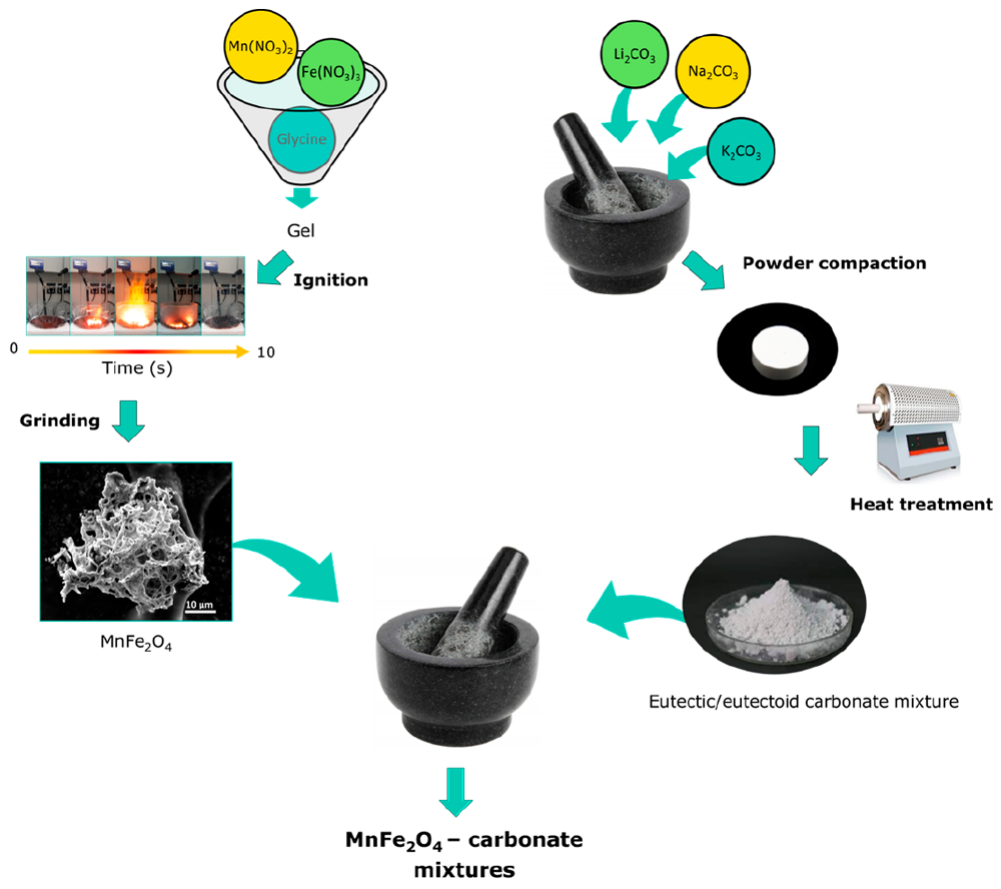
Scheme of the procedure used to prepare the MnFe_2_O_4_-carbonate mixtures.

The procedure used for the preparation of the investigated mixtures is schematized in Figure 1. Highly porous nanostructured MnFe_2_O_4_ was synthesized following the self-combustion method. Fe(NO_3_)_3_·9H_2_O (99%, Alfa Aesar) and Mn(NO_3_)_2_·4H_2_O (99%, Alfa Aesar) were used as precursors, while glycine (C_2_H_5_NO_2_, 98.5%, Sigma-Aldrich) was used as complexant and fuel agent. All the experimental details concerning the synthesis and the characterization of the synthesized oxide can be found in our recent work.^25^

The eutectic or eutectoid alkali carbonates were prepared as follows. The pure carbonates Na_2_CO_3_ (≥99.5%, Sigma-Aldrich), Li_2_CO_3_ (≥99.0%, Sigma-Aldrich), and K_2_CO_3_ (≥99.0%, Sigma-Aldrich) were dried at 200 °C for 3 h and then mixed in the desired molar ratio by means of a mortar. Six grams of the obtained mixture were pressed to obtain a 3 mm-high pellet. To form a fine solid solution, the pellets were heated up to 600 °C under CO_2_ atmosphere, quenched, and ground into fine powders (particle size <200 μm) using a mortar. At these temperatures, the Li_2_CO_3_-Na_2_CO_3_ mixture and the ternary Na_2_CO_3_-Li_2_CO_3_-K_2_CO_3_ eutectic mixtures melted without decomposing.^34,35^ This temperature was also sufficiently high to ensure the eutectoid reaction in the Na_2_CO_3_-Li_2_CO_3_ (0.93:0.07), which according to the phase diagram takes place at around 270 °C (Figure S1).

The mixed alkali carbonate powders were then mixed with the as-synthesized MnFe_2_O_4_ by means of a mortar. The composition of the resulting MnFe_2_O_4_-alkali carbonate mixtures are reported in Table 1, together with the abbreviations used in the text.

**Table 1:** Compositions of the MnFe_2_O_4_-Alkali Carbonate Mixtures Together with the Abbreviation Used in the Text^a^

Composition	Abbreviation	Mass change (1:1)^b^	Mass change (2:3)^c^
MnFe_2_O_4_ + Na_2_CO_3_	Na	13.0 wt %	15.1 wt %
MnFe_2_O_4_ + (Na_0.93_Li_0.07_)_2_CO_3_	7% Li-Na	13.1 wt %	15.3 wt %
MnFe_2_O_4_ + (Na_0.48_Li_0.52_)_2_CO_3_	50% Li-Na	13.7 wt %	16.1 wt %
MnFe_2_O_4_ + (Na_0.33_Li_0.32_K_0.34_)_2_CO_3_	Li-K-Na	12.9 wt %	15.2 wt %

a The theoretical mass changes of the decarbonation reaction for a MnFe_2_O_4_-carbonate ratio of 1:1 (reaction 5) and the WS reaction for a MnFe_2_O_4_-carbonate ratio of 2:3 (reaction 1) are also reported.

b MnFe_2_O_4_ to carbonate ratio 1:1.

c MnFe_2_O_4_ to carbonate ratio 2:3.

### 2.3 Thermal Analysis

#### 2.3.1 Melting and Decomposition Temperature of the Eutectic and Eutectoid Alkali Carbonate Mixtures in the Presence and Absence of MnFe_2_O_4_

The melting and decomposition temperatures were evaluated by thermogravimetry using a STA 449 F3 Jupiter thermobalance (NETZSCH) with a TGA-DSC sensor. Initially, the melting and decomposition temperatures of pure Na_2_CO_3_ and the eutectic or eutectoid alkali carbonate mixtures in the absence of MnFe_2_O_4_, namely C-Na, C-7%LiNa, C-50%Li-Na, and C-Li-K-Na, were determined (Table 2). Around 20 mg of sample was heated up to 900 °C under 20 mL/min of N_2_ with a heating ramp of 10 °C/min. The decomposition temperatures of pure Na_2_CO_3_ and the eutectic or eutectoid alkali carbonate mixtures in the presence of MnFe_2_O_4_, namely Na, 7% LiNa, 50% Li-Na, and Li-K-Na, were determined using an analogous procedure.

**Table 2:** Compositions of the Different Alkali Carbonate Mixtures Together with the Abbreviation Used in the Text

Composition	Abbreviation
Na_2_CO_3_	C-Na
(Na_0.93_Li_0.07_)_2_CO_3_	C-7% Li-Na
(Na_0.48_Li_0.52_)_2_CO_3_	C-50% Li-Na
(Na_0.33_Li_0.32_K_0.34_)_2_CO_3_	C-Li-K-Na

#### 2.3.2 Reversibility under Nonoxidative Conditions

The decarbonation-carbonation reversibility of the MnFe_2_O_4_-alkali carbonate mixtures (Table 1) was evaluated by thermogravimetry using a TGA-DSC1 LF equipment from Mettler Toledo. A MnFe_2_O_4_-to-carbonate ratio of 1:1 was used to test the decarbonation-carbonation reaction:
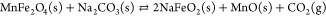
5For each measurement, 10 mg of sample was used. Each sample was initially heated from 50 to 750 °C and then kept at isothermal conditions for 2 h under N_2_ atmosphere (60 mL/min). This step enabled activation of the material and reduced any potential microstructural differences among the samples. The reversibility of the mixtures was tested under dynamic conditions in the 500–750 °C temperature range. For each cycle, the sample underwent a cooling segment (carbonation) from 750 to 500 °C under 60 mL/min of CO_2_, followed by a heating segment (decarbonation) from 500 to 750 °C under 60 mL/min of N_2_. In both cases, a heating/cooling rate of 10 °C/min was used. These two steps were repeated 20 times. To better evaluate the effect of carbonate composition, the thermograms of the four mixtures were analyzed to identify different parameters such as (i) the CO_2_ desorbed (wt %); (ii) the temperature at which 1% of the CO_2_ is released (*T*_onset_); (iii) the temperature at which 90% of the CO_2_ is released (*T*_offset_); the difference between the *T*_offset_ and the *T*_onset_ (Δ*T*), which gives a measure of the reaction rate.

The accuracy of the measurements was verified by testing calcium oxalate monohydrated as a reference standard. This compound undergoes dehydration when heated under an inert atmosphere. The theoretical mass change during this reaction is 12.33 wt %, which is close to the mass changes studied in this work. A mass loss of 12.23 wt % was determined experimentally. Accordingly, the error in the CO_2_ quantification is below 1%.

#### 2.3.3 Hydrogen Production Cycles under Oxidative Conditions

**Figure 2:**
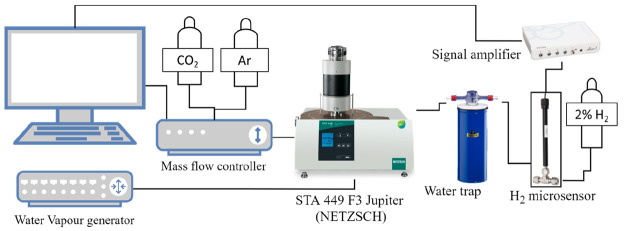
Scheme of the TGA setup used for the H_2_ production experiments.

The hydrogen production under oxidative conditions of the MnFe_2_O_4_-alkali carbonate mixtures (Table 1) was studied in a STA 449 F3 Jupiter thermobalance (NETZSCH) with a resolution of 0.1 μg coupled to a water vapor generator provided by aDROP GmbH and an H_2_-NPLR needle microsensor (UNISENSE A/S, Denmark). A schematic representation of the experimental setup is reported in Figure 2. In accordance with the stoichiometry of reaction 1, a MnFe_2_O_4_-to-carbonate ratio of 2:3 was used. The mixtures were submitted to five consecutive thermochemical cycles consisting of two reaction steps: a first WS step (reaction 1) followed by a reduction step (reaction 2). Before the first cycle, the reaction mixture was heated up to 750 °C under 100 mL/min of N_2_ with a heating ramp of 10 °C/min. Later, in the WS step, the mixture reacted at 750 °C in the presence 100 mL/min of N_2_ and 0.5 g/h of water vapor. The WS step lasted for 5 h for the first cycle and for 3 h for the remaining 4 cycles. In fact, preliminary experiments highlighted that the WS reaction was slower during the first cycle. After the WS step, the water supply was immediately stopped, while the N_2_ flow was maintained for another 5 min to remove the remaining H_2_O from the reaction chamber. The reduction reaction was carried out at the same temperature, i.e., 750 °C, in the presence of 100 mL/min of CO_2_. Each thermochemical cycle lasted approximately 24 h. The obtained thermograms were corrected in order to suppress the buoyancy effects due to the variation in the density and flow of the reactive gases.

The H_2_ concentration in the exhaust gases during the WS step was monitored by means of the H_2_-NPLR microsensor. Before every WS step, the H_2_ sensor was calibrated by using a two-point calibration as recommended by the provider. To this aim, pure N_2_ and an N_2_ with 2 vol % H_2_ standards were used.

### 2.4 Structural and Microstructural Characterization

The studied mixtures were characterized by X-ray diffraction (XRD) analysis using a Bruker D8 Discover equipped with a LYNXEYE XE detector and a monochromatic Cu Kα1 radiation source of λ = 1.54056 Å. The collected patterns were then analyzed according to the Rietveld method^36^ by using the MAUD software.^37^ The effect of cycling on the powder morphology was analyzed by scanning electron microscopy (SEM) using a Quanta 200 FEG (FEI Company, Hillsboro, OR, USA) operating in high vacuum mode.

### 2.5 Hot-Stage Optical Microscopy

Hot-stage optical microscopy was used to follow the microstructural changes in some selected mixtures at increasing temperatures using a Zeiss Axioscope 5MAT microscope equipped with an Axiocam 208 Color camera. A LINKAM TS1500 hot stage was used to heat the sample under a controlled atmosphere (CO_2_). To obtain a flat surface that enabled a good focus during the imaging, around 50 mg of MnFe_2_O_4_ were pressed into pellets with a 5 mm diameter and 1 mm height by using a manual pelletizer. In this way, the low applied load did not affect the porous structure of the original MnFe_2_O_4_ powders. The obtained pellet was located inside a cylindrical alumina crucible and placed in the LINKAM hot stage. Carbonate powders (either pure Na_2_CO_3_ or (Na_0.93_Li_0.07_)_2_CO_3_) were deposited on top of the pellet. A few milligrams of the selected carbonate powders were dispersed in ≈1 mL of acetone and then dropped on the pellet using a syringe. The procedure was optimized to obtain a good distribution of the carbonate particles without agglomeration. The samples were heated under CO_2_ atmosphere (50 mL/min) at 10 °C/min until the surface of the pellet reached ≈750 °C, kept in isothermal conditions for 10 min, and then cooled at 5 °C/min. To compensate for any temperature gradient between the LINKAM thermocouple (side of the crucible) and the top of the pellet surface, a temperature calibration was initially performed. A MnFe_2_O_4_ pellet (without carbonate particles) was heated up at 10 °C/min from room temperature to 1000 °C while measuring the pellet surface temperature through the top quartz window of the LINKAM hot stage using a pyrometer (CEIA). The same gas flow used during the experiments was used. The calibration curve is reported in Figure S10.

## 3 Results and Discussion

### 3.1 Melting and Decomposition Temperature of the Eutectic or Eutectoid Alkali Carbonate Mixtures in the Presence and Absence of MnFe_2_O_4_

The melting and decomposition temperatures of the pure Na_2_CO_3_ and the eutectic or eutectoid alkali carbonate mixtures in the absence and presence of MnFe_2_O_4_ were evaluated by thermogravimetry. The thermograms and DSC profiles of the samples without MnFe_2_O_4_, i.e., C-Na, C-7% LiNa, C-50% Li-Na, and C-Li-K-Na, are depicted in the Figure S2, while the melting and decomposition temperatures are shown in the Table S1. All the eutectic or eutectoid alkali carbonate mixtures exhibited a lower melting temperature and decomposition temperature than pure Na_2_CO_3_.

Analogous experiments were carried out in the presence of MnFe_2_O_4_, i.e., Na, 7% LiNa, 50% Li-Na, and Li-K-Na mixtures. The thermograms and DSC profiles are depicted in Figure S3, while the decomposition temperatures are shown in Table S2. The decomposition of the alkali carbonates seems to occur in several steps in the presence of MnFe_2_O_4_. As expected, all the MnFe_2_O_4_-eutectic or eutectoid alkali carbonate mixtures exhibited a lower decomposition temperature than the MnFe_2_O_4_-Na_2_CO_3_ mixture (Na).

To confirm the above-mentioned results, theoretical calculations were carried out using the FactSage program. The molar fraction of the different chemical species and their corresponding thermodynamic phases found at different temperatures under an inert atmosphere are depicted in Figure S4. The theoretical decarbonation temperatures obtained by thermodynamic calculations are summarized in Table S2. For the Na mixture, the reaction is predicted to be favored at temperatures higher than 358 °C; the conversion increases with the temperature, and at temperatures higher than 650 °C only the products of the reaction 5 are predicted to be stable. For both 7% LiNa, 50% Li-Na, a first decarbonation reaction is predicted to be thermodynamically favored already at 279 °C, due to the reaction of Li_2_CO_3_ with MnFe_2_O_4_ to form LiFeO_2_ and MnO. The reaction between Na_2_CO_3_ and the remaining MnFe_2_O_4_ is favored only at temperatures higher than 499 and 580 °C for 7% Li-Na and 50% Li-Na, respectively. Finally, for Li-K-Na, three separate decarbonation reactions are predicted to take place at temperatures of 196, 478, and 717 °C.

The temperature values obtained by equilibrium calculations were lower than the experimental values (Table S2). This is expected, as the theoretical values are obtained by equilibrium calculations that do not consider kinetic limitations that can lead to metastable phases during the experimental measurement. In particular, kinetics constraints can arise from (i) the activation energies of chemical reactions, diffusion processes, etc., and (ii) the experimental conditions, which may not allow the system to reach thermodynamic equilibrium. In both cases, this can lead to metastable states, especially at low temperatures. In fact, the difference between experimental and equilibrium values (Table S2) is bigger at low temperatures, where kinetic constraints are more pronounced.

Considering these limitations, the theoretical results are in line with the experimental results. For all the Li-containing mixtures, the release of CO_2_ is thermodynamically favored at lower temperatures compared to the Na mixture due to the formation of LiFeO_2_.

### 3.2 Reversibility under Nonoxidative Conditions

**Figure 3:**
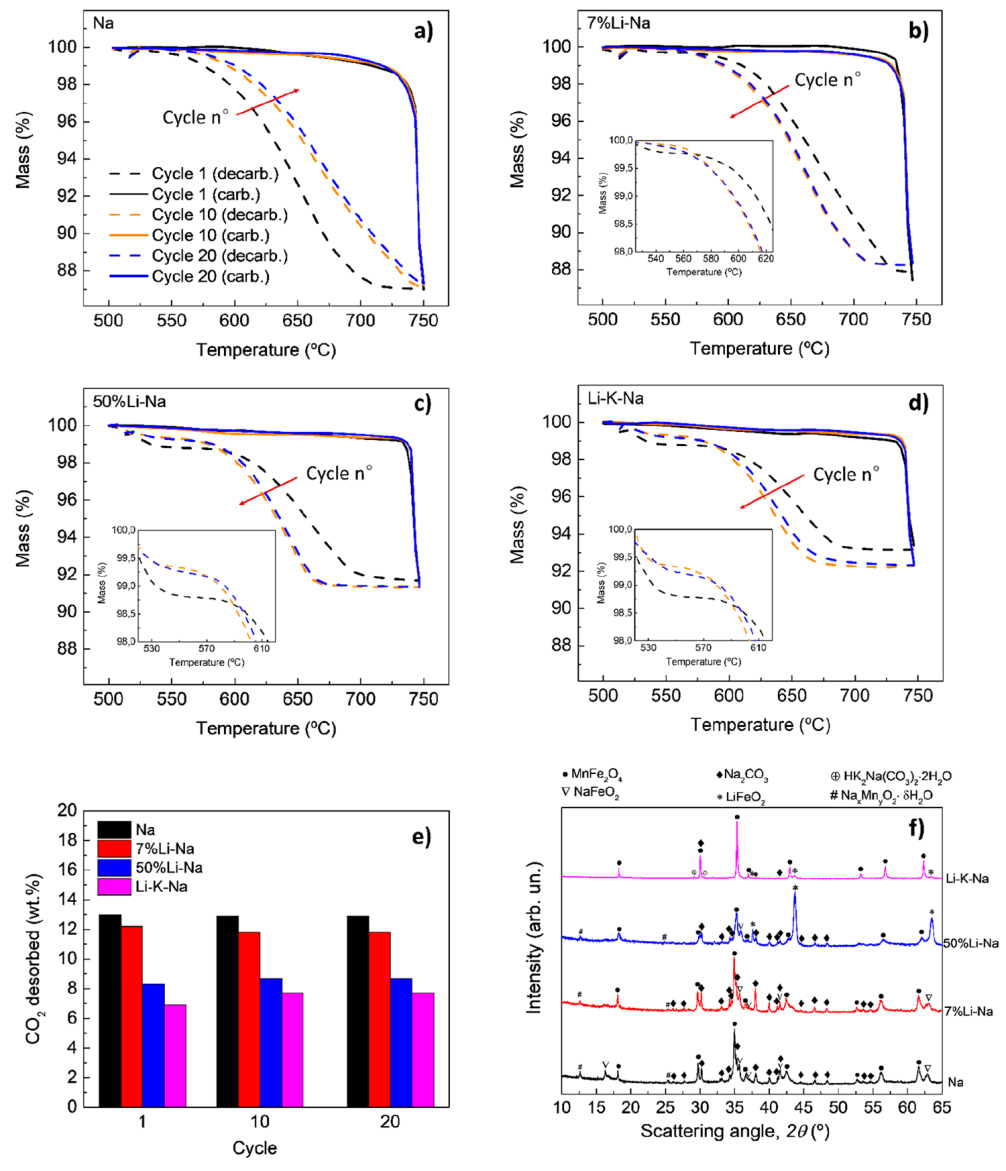
(a–d) Decarbonation (dotted line) and carbonation (full line) thermograms of the Na, 7% Li-Na, 50% Li-Na, and Li-K-Na mixtures during 20 decarbonation-carbonation cycles performed in dynamic conditions between 500 and 750 °C. Red arrows indicate the increase of cycle number, i–e, from cycle 1 to 20. (e) Amount of CO_2_ desorbed (wt %) by the different mixtures during 20 decarbonation-carbonation cycles. For the sake of simplicity, only cycle nos. 1 (black), 10 (orange), and 20 (blue) are compared for each mixture. (f) XRD analysis of the cycled mixtures.

In this section, the reversibility of the different MnFe_2_O_4_-alkali carbonate mixtures was investigated in the absence of water for 20 decarbonation-carbonation cycles (reaction 5). The mass profiles and the amount of CO_2_ desorbed (wt %) for the first, tenth, and twentieth cycles are shown in Figure 3. The XRD analysis after the 20 cycles is also reported.

In the first cycle, the amount of CO_2_ desorbed follows the order Na > 7% Li-Na > 50% Li-Na > Li-K-Na, with measured mass losses of 13, 12.2, 8.3, and 6.9 wt %, respectively. These values did not significantly vary during the 20 cycles and correspond to 100%, 93%, 60%, and 53% of the theoretical values calculated for the different mixtures (Table 1). These reversibility differences align with the XRD analysis performed on the four mixtures after 20 cycles (Figure 3f). Indeed, the cycled Na mixture showed almost complete regeneration of the MnFe_2_O_4_ and Na_2_CO_3_, with a small amount of residual NaFeO_2_. A few remaining Bragg peaks match with a hydrated Na-Mn oxide named Birnessite (COD 1531679). This also was present in the other mixtures and is likely due to the partial hydration/oxidation of the mixtures before the XRD analysis were performed. As the Birnessite can accommodate different amounts of H_2_O to form a broad range of nonstoichiometric layered oxides, we here refer to a general formula Na_*x*_Mn_*y*_O_2_·δH_2_O.

Compared to the Na mixture, the cycled 7% Li-Na did not show appreciable differences in terms of phase composition, probably due to the relatively small percentage of Li atomic substitution. No differences could be appreciated also in the average lattice parameter of the MnFe_2_O_4_ phase, as both cycled Na and 7% Li-Na showed values of 8.50 Å.

On the other hand, the cycled 50% Li-Na showed a significant amount of LiFeO_2_ (COD 1541312), which explains the lower CO_2_ capacity of the mixture. Finally, the low reversibility of Li-K-Na can be explained by the formation of mixed Na-K carbonate. More details are reported in Section S4.1.

**Figure 4:**
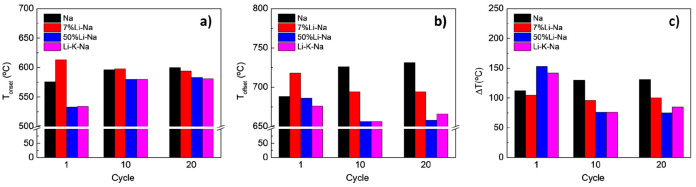
(a) *T*_onset_, (b) *T*_offset_, and (c) Δ*T* (*T*_offset_ – *T*_onset_) of the four mixtures during cycle nos. 1, 10, and 20. *T*_onset_ refers to the 1 wt % loss, while *T*_offset_ to 90% of the total mass loss during the cycle.

Apart from the CO_2_ capacity, the carbonate composition also influenced the reaction kinetics, especially that of the decarbonation step (Figure 3a–d). This can be better appreciated by looking at the values of *T*_onset_, *T*_offset_, Δ*T*, which are reported in Table 3 and plotted in Figure 4a–c. Experimental details about the procedure to evaluate these parameters can be found in section 2.3.2. The decarbonation kinetics of the reference Na sample worsened upon cycling as can be observed by the right shift in the profiles (Figure 3a). As a result, both the *T*_onset_ and the *T*_offset_ increased (Figure 4a, b), as well as the Δ*T* (Figure 4c). This worsening can be attributed to the sintering and coalescence of the material upon cycling.^25^

**Table 3:** CO_2_ Desorbed, *T*_onset_, *T*_offset_, and Δ*T* (*T*_offset_ – *T*_onset_) Obtained for the Four Investigated Mixtures During 20 Decarbonation-Carbonation Cycles^a^

	Na				7% Li-Na			
*N*°_cycle_	CO_2_desorbed (wt %)	*T*_onset_ (°C)	*T*_offset_ (°C)	Δ*T*	CO_2_desorbed (wt %)	*T*_onset_ (°C)	*T*_offset_ (°C)	Δ*T*
1	13	576	688	112	12.2	613	718	105
10	12.9	596	726	130	11.8	598	694	96
20	12.9	600	731	131	11.8	594	694	100

	50% Li-Na				Li-K-Na			
*N*°_cycle_	CO_2_desorbed (wt %)	*T*_onset_ (°C)	*T*_offset_ (°C)	Δ*T*	CO_2_desorbed (wt %)	*T*_onset_ (°C)	*T*_offset_ (°C)	Δ*T*
1	8.3	533	686	153	6.9	534	676	142
10	8.7	580	656	76	7.7	580	656	76
20	8.7	583	658	75	7.7	581	666	85

a *T*_onset_ refers to the 1 wt % loss, while *T*_offset_ refers to 90% of the total mass loss during the cycle.

The opposite behavior was observed for the three mixed carbonates as the CO_2_ desorption profiles shifted to lower temperatures during the cycling. The *T*_offset_ and Δ*T* of the three mixed carbonates decreased during the cycles. Moreover, after 20 cycles the three mixed carbonates showed lower *T*_offset_ and Δ*T* than the Na reference mixture. However, it is worth considering that the mixed carbonates showed lower CO_2_ desorption than Na, thus a direct comparison is not so straightforward as a higher mass loss may have needed more time. This behavior could be attributed to the fact that the initial MnFe_2_O_4_-alkali carbonate mixtures were not fully homogeneous. The successive melting and solidification of the carbonate mixtures upon cycling could have led to a more homogeneous mixture resulting in faster decarbonation kinetics upon cycling.

A closer look at the first part of the decarbonation profiles allows one to appreciate more differences among the mixtures (see boxes in Figure 3b–d). While Na lost mass monotonically, the mixed carbonate mixtures exhibited a two-step mechanism. This is particularly evident in the 50%-LiNa and Li-K-Na mixtures and can be appreciated by looking at the small boxes in Figure 3c, d. This is in line with the TG-DSC data previously presented for the different mixtures (see section 3.1 and Supporting Information). Due to this two-step mechanism, the *T*_onset_ of the two mixtures tended to increase with the number of cycles but with values lower than the reference Na mixture. Even the 7% Li-Na exhibited a two-step desorption during the first cycle, but this behavior disappeared upon cycling and the two steps merged into one. As result, the *T*_onset_ of the 7% Li-Na slightly decreased with the number of cycles.

A common feature among the four mixtures is that only small changes are appreciated between cycles 10 and 20, which suggests that the microstructure stabilized within the first 10 cycles.

Despite all these differences in the decarbonation kinetics, the carbonation reaction was not significantly affected by the carbonate composition and the number of cycles. In all cases, the reaction is very fast; the carbon dioxide uptake needed around 3 min in the range of 750 and 720 °C.

Under nonoxidative conditions, the 7% Li-Na mixture showed the best performance among the three mixed carbonates. In fact, it showed a CO_2_ desorption capacity that was only slightly lower than the reference Na mixture, but with significantly better decarbonation kinetic and lower maximum required temperature. This is particularly evident when comparing the two mixtures after 20 cycles (see Table 3). The 7% Li-Na showed a *T*_offset_ and a Δ*T* that are 34 and 31 °C, respectively, lower than those of the Na mixture.

### 3.3 Hydrogen Production Cycles under Oxidative Conditions

The reversibility and the WS reaction kinetics of the different MnFe_2_O_4_-alkali carbonate mixtures were investigated in the presence of water. For that purpose, the Na, 7% Li-Na, 50% Li-Na, and Li-K-Na mixtures were submitted to five consecutive thermochemical cycles under oxidative conditions consisting of two reaction steps, the WS (reaction 1) and the RE step (reaction 2). Experimental details are reported in Section 2.3.3. During the WS step, CO_2_ is released, water is reduced to form H_2_, while oxygen from water is incorporated in the lamellar NaFe_2/3_Mn_1/3_O_2_. This generates a consequent reduction of the overall mass of the mixtures (reaction 1). In contrast, during the RE step, the mixtures experiment a mass gain due to their reaction with CO_2_, flanked by a small mass loss due to O_2_ release (reaction 2). For this reason, the mass profile can give information about the reversibility of the overall process. The theoretical mass changes of the four mixtures are reported in Table 1.

**Figure 5:**
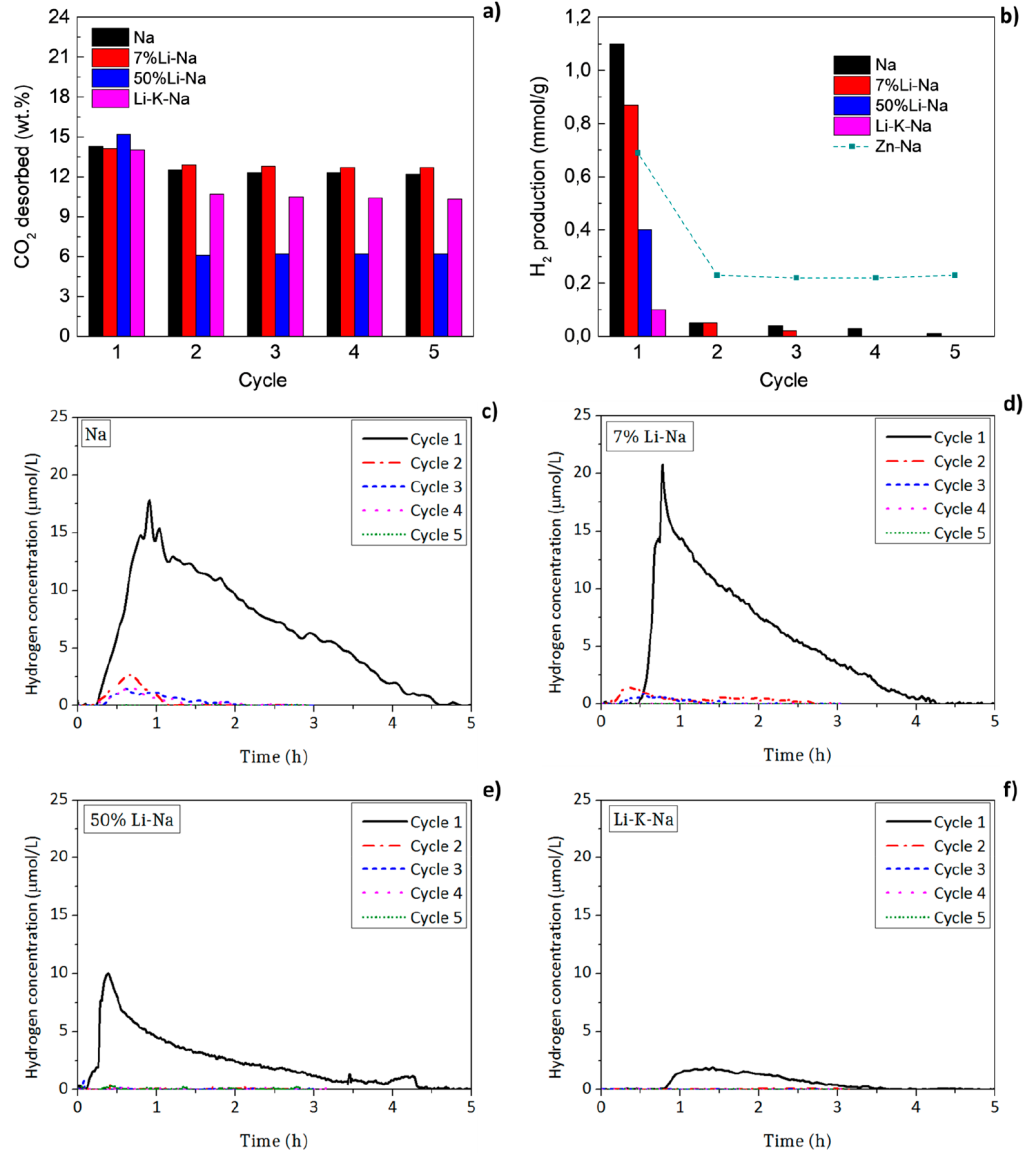
(a) Amount of CO_2_ desorbed (wt %) and (b) of H_2_ produced (mmol/g) by the four mixtures during 5 consecutive cycles at 750 °C. The H_2_ produced under the same experimental conditions by the 2Mn_0.95_Zn_0.05_Fe_2_O_4_-3Na_2_CO_3_ mixture (Zn-Na) is also reported as a term of comparison.^25^ Reproduced with permission from ref (25). Copyright 2022 Elsevier. (c–f) H_2_ concentration (micromoles per liter of carrier gas) detected in the exhaust gases of the STA during the WS step of the four investigated mixtures.

The hydrogen production of each of the five consecutive cycles for the different mixtures are reported in Table 4 and are plotted in Figure 5. For the sake of brevity, thermograms obtained for the four investigated mixtures over 5 thermochemical cycles are reported in Figure S5. The reproducibility of the hydrogen production measurements was assessed by replicating the first and second cycles for the 7% Li-Na mixture. Two replicates of the first cycle were carried out while only one replicate was carried out in the case of the second cycle due to the long duration of the experiments. The results of the replicates are reported in Table S3 and the evolution of the hydrogen concentration in the exhaust gases for the replicates of the first cycle are depicted in Figure S6. In the case of the first cycle, a mean value of 0.83 ± 0.04 mmol H_2_/g was observed, which indicates that the hydrogen measurements are reproducible.

**Table 4:** Mass Changes and H_2_ Production of the Four Different Mixtures during Five Consecutive Cycles^a^

	Na			7% Li-Na		
*N*°_cycle_	Mass loss (wt %)	Mass gain (wt %)	H_2_production (mmol/g)	Mass loss (wt %)	Mass gain (wt %)	H_2_production (mmol/g)
1	14.3	12.7	1.10	14.1	13.1	0.87
2	12.5	12.4	0.05	12.9	12.8	0.05
3	12.3	12.3	0.04	12.8	12.8	0.02
4	12.3	12.2	0.03	12.7	12.7	–
5	12.2	11.3	0.01	12.7	11.8	–
			Total: 1.24			Total: 0.94

	50% Li-Na			Li-K-Na		
*N*°_cycle_	Mass loss (wt %)	Mass gain (wt %)	H_2_production (mmol/g)	Mass loss (wt %)	Mass gain (wt %)	H_2_production (mmol/g)
1	15.2	5.4	0.4	14.0	9.1	0.1
2	6.1	6.1	–	10.7	10.4	–
3	6.2	6.1	–	10.5	10.2	–
4	6.2	6.1	–	10.4	10.1	–
5	6.2	6.2	–	10.3	9.9	–
			Total: 0.4			Total: 0.1

a Mass loss refers to the water splitting (WS) step and mass gain to the reduction step. The MnFe_2_O_4_-to-carbonate ratio is 2:3.

To evaluate the reversibility of the different mixtures upon cycling, the mass changes and the hydrogen production were assessed (Table 4). As previously observed under nonoxidizing conditions, the Na and 7% Li-Na mixtures showed the highest reversibility in terms of mass change, i.e., CO_2_ desorption/capture. During the first cycle, the two mixtures desorbed 14.3 and 14.1 wt %, respectively, which corresponds to 94.7% and 92.1% of their theoretical CO_2_ capacities. Only a small loss of reversibility in terms of mass change was observed after the first cycle for the two mixtures (Figure 5a). Moreover, in the last water-splitting cycle, the 7% Li-Na showed a slightly higher mass loss (83.0% of the theoretical value) compared to Na (80.1% of the theoretical value). Conversely, the 50%Li-Na showed completely different behavior. During the first cycle, it desorbed 15.2 wt % of CO_2_ (94.4% of the theoretical CO_2_ capacity), but this value then dropped to ≈6 wt % for the second cycle, whose value corresponds to ≈38% of the theoretical CO_2_ capacity. Even the Li-K-Na mixture showed a significant loss of reversibility in terms of mass change between the first (14 wt %) and the second cycle (10.7 wt %). Nonetheless, its reversibility was significantly better than that of the 50% Li-Na. In this regard, it is worth mentioning that under nonoxidizing conditions the latter showed better performances than the Li-K-Na.

**Figure 6:**
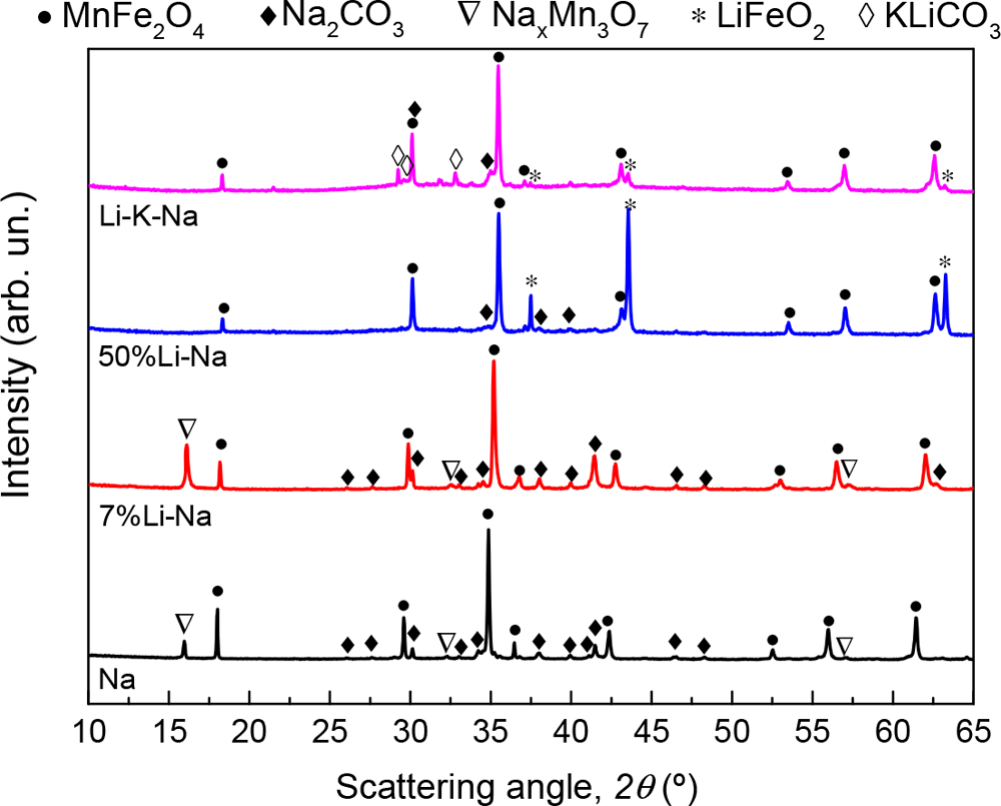
XRD analysis of the four mixtures after 5 H_2_ production cycles performed under isothermal conditions at 750 °C.

The differences among the mixtures were even more evident when comparing the H_2_ productions (Table 4 and Figure 5b). However, a general trend was also observed: all the mixtures produced hydrogen in the first cycle, while little or no hydrogen was produced in successive cycles. In particular, the 50% Li-Na and Li-K-Na exhibited no reversibility in terms of hydrogen production, which can be attributed to the irreversible formation of LiFeO_2_ (Figure 6). More details are reported in section S4.2. The best results were obtained for the Na mixture, with a hydrogen production of 1.10 mmol H_2_/g in the first cycle. In the second cycle, this value dropped to 0.05 mmol/g and eventually was reduced to 0.01 mmol/g in the fifth cycle. This drop in the H_2_ production is reflected by the presence of the nonstoichiometric Na_*x*_Mn_3_O_7_ phase in the XRD pattern (Figure 6). The 7% Li-Na mixture exhibited lower reversibility in terms of hydrogen production but still significantly higher than the 50% Li-Na and Li-K-Na mixtures. During the first cycle, the 7% Li-Na mixture produced 0.87 mmol H_2_/g, which was higher than the 0.69 mmol H_2_/g recently observed under the same experimental conditions for a Zn-doped Na mixture.^25^ However, the amount of H_2_ produced by the 7% Li-Na mixture dropped down to 0.05 mmol H_2_/g in the second cycle and to 0.02 mmol/g during the third one, while no hydrogen production was detected in the last two cycles. In this case, the loss of reversibility can also be ascribed to the formation of the nonstoichiometric Na_*x*_Mn_3_O_7_ phase. Although a quantitative analysis cannot be provided, the amount of this phase was more evident than in the Na mixture (Figure 6) as can be appreciated by the higher intensity of the main peak at 2θ ≈ 18.

The amounts of H_2_ produced by the Na and 7% Li-Na mixtures in the first WS are in line with the values reported in previous works for the same thermochemical cycle.^25,28,29,^ For instance, Murmura et al.^29^ performed WS tests at 750 °C under different H_2_O partial pressure conditions and reported H_2_ yields between 83 and 75%. Considering that the theoretical H_2_ yield of the thermochemical cycle is 1.28 mmol/g, this corresponds to H_2_ productions between 1.06 and 0.96 mmol/g. In another work,^28^ the same research group reported a quite stable H_2_ production during 25 cycles at 750 °C. In particular, a reactive pellet of MnFe_2_O_4_-Na_2_CO_3_ was still able to produce ≈0.15 mmol/g after 25 cycles. In the same conditions, a more conventional MnFe_2_O_4_-Na_2_CO_3_ mixture prepared by ball milling quickly lost reversibility. In fact, despite the H_2_ produced during the first cycle being higher than the one observed for the reactive pellet (0.7 mmol/g vs 0.25 mmol/g), during the second cycle its production dropped to ≈0.05 mmol/g.^28^ Interestingly, this value perfectly matches the H_2_ produced by Na and 7%Li-Na during the second cycle. Unfortunately, no data were reported for further cycling of the conventional mixture; however, the authors reported that “in the third cycle, hydrogen production has almost vanished and the sample is considered unable to further perform cycles in the used time-temperature conditions.” The authors attributed the best performance of the reactive pellet compared to the conventional mixture to the fine intermixing between MnFe_2_O_4_ and Na_2_CO_3_, which could be achieved by their proposed new methodology. The importance of the initial powder’s morphology has been confirmed by a recent work.^38^ In particular, coprecipitated MnFe_2_O_4_ produced approximately 30% more H_2_ than ball-milled MnFe_2_O_4_, also ensuring a higher reversibility during three cycles.

On the one hand, these considerations suggest that the reversibility loss observed for the Na and 7% Li-Na mixtures in this work could be attributed to a nonoptimal initial morphology of the mixture. However, in our previous work^25^ a Zn-doped Na mixture prepared following the same procedure, showed good stability upon cycling under the same experimental conditions (see Figure 5b). Thus, it is reasonable that more things can cause the loss of reversibility of the thermochemical cycle in question. In this regard, a direct comparison with results obtained by other groups can be very tricky due to all the possible experimental differences related to materials preparation, presence of impurities, experimental conditions, geometry of the reactor, etc.

To determine the reasons behind the hydrogen production drop of the 7% Li-Na mixture, XRD analysis of the mixture was performed after the first and second WS reaction steps (Figure S7). In both cases, NaMn_1/3_Fe_2/3_O_2_ was detected as the main phase, followed by minor amounts of unreacted Na_2_CO_3_, and MnFe_2_O_4_. However, the XRD analysis did not indicate the presence of nonreversible thermodynamically stable phases that could explain this drop in hydrogen production (i.e., LiFeO_2_). Even the SEM analysis of the 7% Li-Na mixture after the first and second WS reaction steps did not highlight significant morphological variations (Figure S8, Supporting Information). These results suggest that the 7% Li-Na and the Na mixtures share the same deactivation process that causes the drop in hydrogen production after the first cycle and the complete loss of reversibility in the following few cycles. Such loss of reversibility is likely due to the incomplete reduction of Mn^3+^ to Mn^2+^ during the RE step, which results in the formation of the nonstoichiometric Na_*x*_Mn_3_O_7_ phase.^25^ The loss of reversibility due to the formation of similar nonstoichiometric phases has already been reported by another group.^26^ This can be attributed to the loss of reactive surfaces in the material due to sintering and coalescence phenomena taking place upon cycling at high temperatures. As a result, the Na-deintercalation is limited and the O_2_ release is hindered.

**Figure 7:**
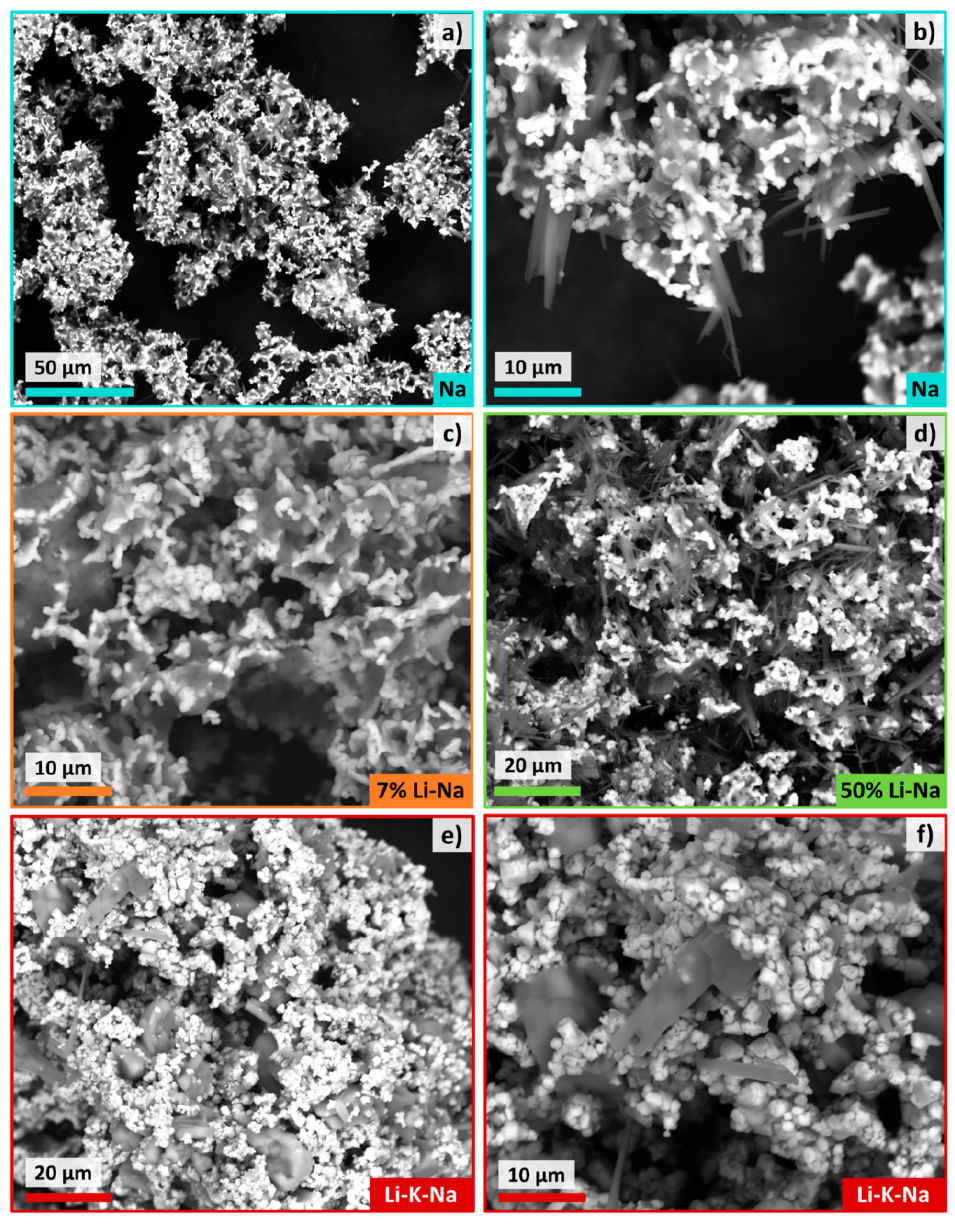
SEM images of the four mixtures after 5 H_2_ production cycles under isothermal conditions at 750 °C. Backscattered electron (BSE) images of (a, b) Na, (c) 7% Li-Na, (d) 50% Li-Na, and (e, f) Li-K-Na samples are reported.

For this reason the cycled mixtures were analyzed by SEM (Figure 7). Backscattered electron imaging of the Na mixture (Figure 7a) highlighted the presence of areas rich in lighter elements (darker areas) that can be ascribed to Na_2_CO_3_, while the brighter areas suggest the presence of substances containing heavier elements, likely MnFe_2_O_4_. A closer look reveals the presence of needle-shaped crystals (Figure 7b) that can be attributed to the partial hydration of Na_2_CO_3_ to form thermonatrite due to the contact with moisture before the SEM analysis was performed.^25^ The 7% Li-Na shows a similar morphology and phase distribution of Na (Figure 7c) but without the presence of needle-shaped thermonatrite. This is somehow unexpected as all the mixtures were stored in the same way, and similar needle-shaped crystals can be found in the 50% Li-Na mixture (Figure 7d), and in less abundance, in the Li-K-Na. In any case, the 7% Li-Na mixture appears more sintered and coalesced than Na. This behavior is even more evident in Li-K-Na mixture (Figure 7e, f). This is likely an undesired effect of the lower-melting point of the eutectic and eutectoid carbonate mixtures compared to pure Na_2_CO_3_, as the formation of liquid phases is known to promote the sintering.^25^ The equilibrium calculations of the four mixtures under CO_2_ support this explanation (Figure S9). Indeed, at 750 °C both 50% Li-Na and Li-K-Na show liquid carbonate as the stable phase, which is in line with the higher sintering degree observed for these phases. Such an effect is less evident for the 7% Li-Na mixture, where only part of the carbonate is predicted to be liquid. Thus, the slightly faster loss of reversibility observed for 7% Li-Na compared to Na can be reasonably explained by the partial formation of liquid carbonate, which represents an undesired effect of the atomic substitution strategy adopted.

### 3.4 Kinetics Considerations on the Liquid Carbonate Formation

**Figure 8:**
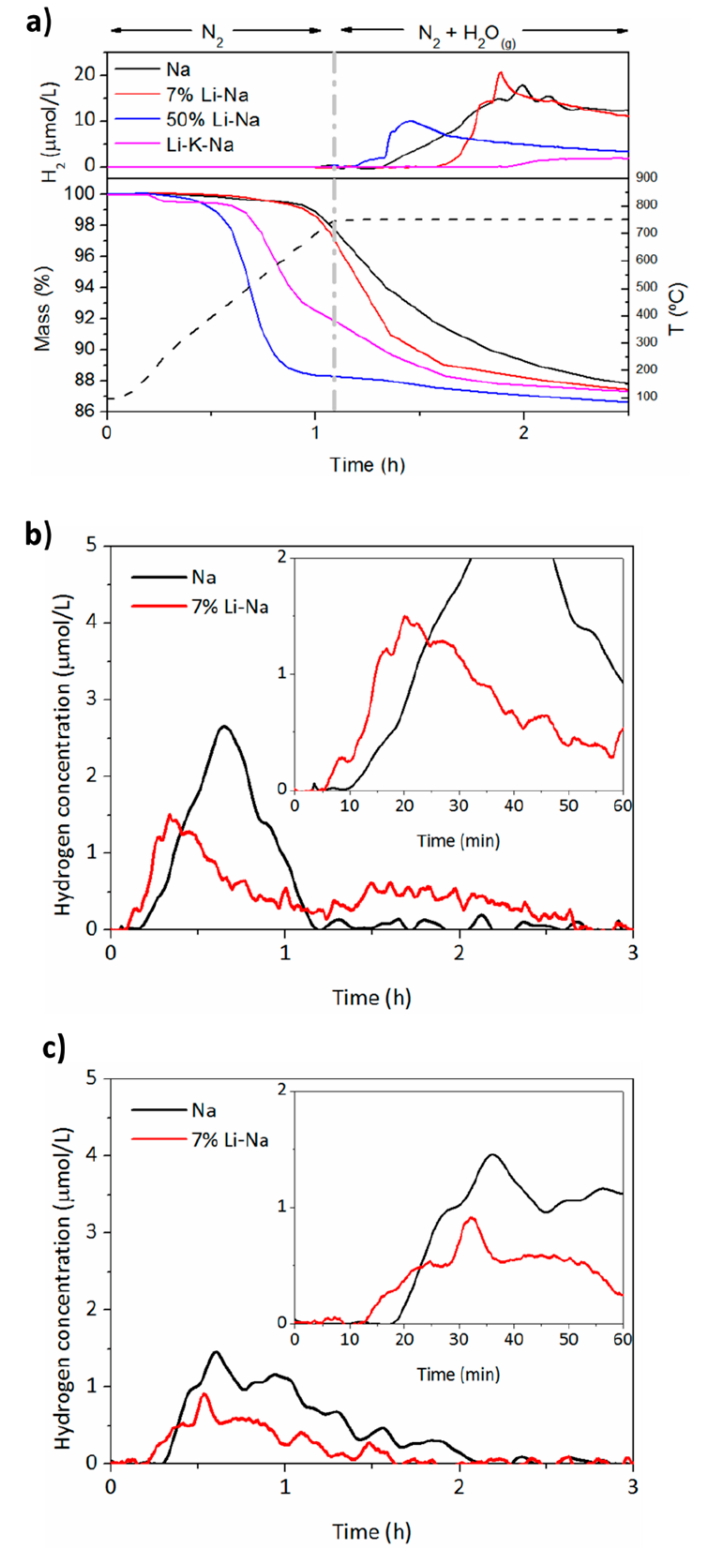
(a) Thermograms and the corresponding evolution of the hydrogen concentration in the exhaust gases during the heating and the first WS step at 750 °C for the four mixtures. The temperature profile is also reported (dashed line). Evolution of the H_2_ hydrogen concentration in the exhaust gases for the Na (black) and 7% Li-Na (red) mixtures for the 2nd (b) and 3rd (c) cycles at 750 °C.

The composition of the carbonates affected the reaction kinetics (Figure 8). For the Li-K-Na mixture, a small mass loss was observed at the beginning of the heating step (*T* < 250 °C). This can be attributed to the release of a small amount of water (≈ 0.5 wt %) and is due to the highly hygroscopic nature of K_2_CO_3_, which quickly absorbs moisture from the air during material sample handling.

On the one hand, the mass profiles in Figure 8a indicate that the decarbonation kinetics during the first dynamic heating under N_2_ were more affected by the percentage of Li content in the alkali carbonate, rather than by the melting temperature of the alkali carbonate. In fact, the Li-K-Na mixture, which has the lower melting point (≈ 400 °C) showed a slower CO_2_ evolution compared to the 50% Li-Na, which melts at around 500 °C. This is supported by thermodynamic calculations (Figure S4), which indicate that during dynamic heating under inert atmosphere, no liquid formation should be expected in any of the four mixtures investigated as the carbonates would decompose before they could melt. Such decomposition is driven by the reaction of the carbonate with MnFe_2_O_4_ (reaction 3), and the driving force is higher in the three mixtures containing Li, as the Li_2_CO_3_ is predicted to decompose at lower temperatures. Kinetics does the rest, as the small dimensions of Li^+^ ions result in faster intercalation to form LiFeO_2_.^15^ However, the H_2_ production kinetics during the following isothermal step at 750 °C do not follow this exact trend, as the time needed to detect H_2_ increases as follows: 50% Li-Na > Na > 7% Li-Na > Li-K-Na (Figure 8a). As the 50% Li-Na and Li-K-Na mixture showed no reversibility, comprehensive kinetic analysis during the cycles cannot be presented for all the mixtures. For this reason, the following discussion will be limited only to the Na and 7% Li-Na mixtures.

Although in the first cycle the WS kinetics for the Na mixture was faster than that of the 7% Li-Na mixture, the opposite behavior was observed for the second cycle (Figure 8b). The WS of the 7% Li-Na mixture started around 5 min before, and the hydrogen production maximum was significantly shifted toward shorter reaction times. This effect seemed to be consistent upon cycling since the same effect was observed for the third cycle (Figure 8c). Such change in terms of kinetics can find an explanation in the microstructural evolution of the two mixtures, which is likely affected by the different experimental conditions that characterize the first and the following cycles, respectively. In fact, the first WS step is preceded by a dynamic heating step, while the following four are always preceded by an isothermal RE step. As said before, during the first heating step alkali carbonates in both the Na and 7% Li-Na mixtures start to decompose before any liquid can form. While for the Na, the reaction follows according to reaction 3, a similar equation can be written for the 7% Li-Na system:
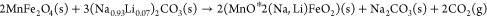
6where (Na,Li)FeO_2_ indicates a fine solid solution of LiFeO_2_ and NaFeO_2_. As Li_2_CO_3_ reacts with MnFe_2_O_4_ at way lower temperatures compared to Na_2_CO_3_, it is reasonable to assume that at the end of the first dynamic heating, the composition of the unreacted carbonate approaches that of pure Na_2_CO_3_, as reported in reaction 6.

In accordance with calculations (Figure S4), in the Na mixture, the formation of the alpha-NaFeO_2_ polymorph starts to be thermodynamically favorable at around 400 °C. On the other hand, in the 7% Li-Na mixture, the formation of this alpha-polymorph is only favorable when the temperature reaches 500 °C. However, kinetics constraints are likely to make this polymorphic transition happen at higher temperatures. Thus, when the dynamic heating step comes to an end and water is introduced in the system, the concentration of alpha-polymorph in the Na mixture is presumably higher than in the 7% Li-Na mixture. As a result, the reaction between MnO*2NaFeO_2_, i.e., MnO*2(Na,Li)FeO_2_ in the case of 7% Li-Na-Na_2_CO_3_, and water vapor to produce H_2_ (Reaction 4) would proceed at a faster rate for the Na mixture than for the 7% Li-Na. This may provide a possible explanation for the faster kinetic observed for the Na compared to 7% Li-Na during the first WS step.

Things are different in the second and third WS steps, as water vapor is introduced right after the carbonates have been regenerated under a CO_2_ atmosphere at 750 °C (RE step, reaction 2). Under such conditions, thermodynamic calculations (Figure S9) indicate that the eutectoid carbonate in the 7% Li-Na is partially in the liquid state, while the Na_2_CO_3_ in the Na mixture remains in the solid state. Hence, the liquid environment of the 7% Li-Na in the second and third WS steps would improve the diffusion of Na and Li toward the MnFe_2_O_4_ resulting in the faster WS kinetics observed for these cycles. This explanation is supported by previous works, where the reaction between Na_2_CO_3_ and SiO_2_ was studied.^39,40^ On the one hand, different reaction mechanisms were observed when heating below the melting point of Na_2_CO_3_ under N_2_ or CO_2_; in the latter case, the decomposition of Na_2_CO_3_ was avoided and the reaction was slower.^39^ However, when the temperature was higher than the melting point of the carbonate, the reaction was accelerated due to the molten carbonate that rapidly wet the SiO_2_ grains.^40^

**Figure 9:**
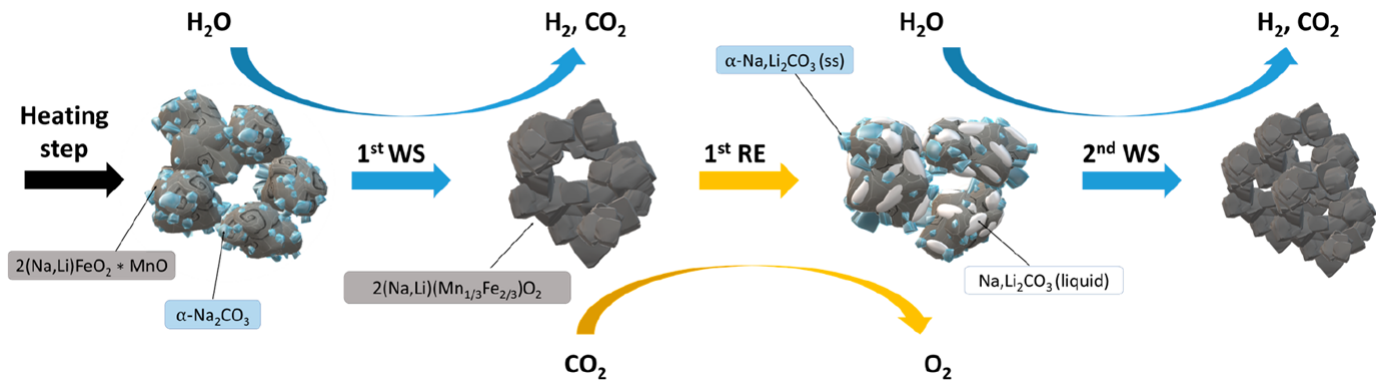
Schematic representation of the microstructural evolution in the 7% Li-Na mixture during the 1st water splitting (WS), the 1st reduction (RE) step, and the 2nd WS step performed under isothermal conditions at 750 °C.

The microstructural evolution proposed for the 7% Li-Na mixture from the first to second WS steps is schematized in Figure 9.

**Figure 10:**
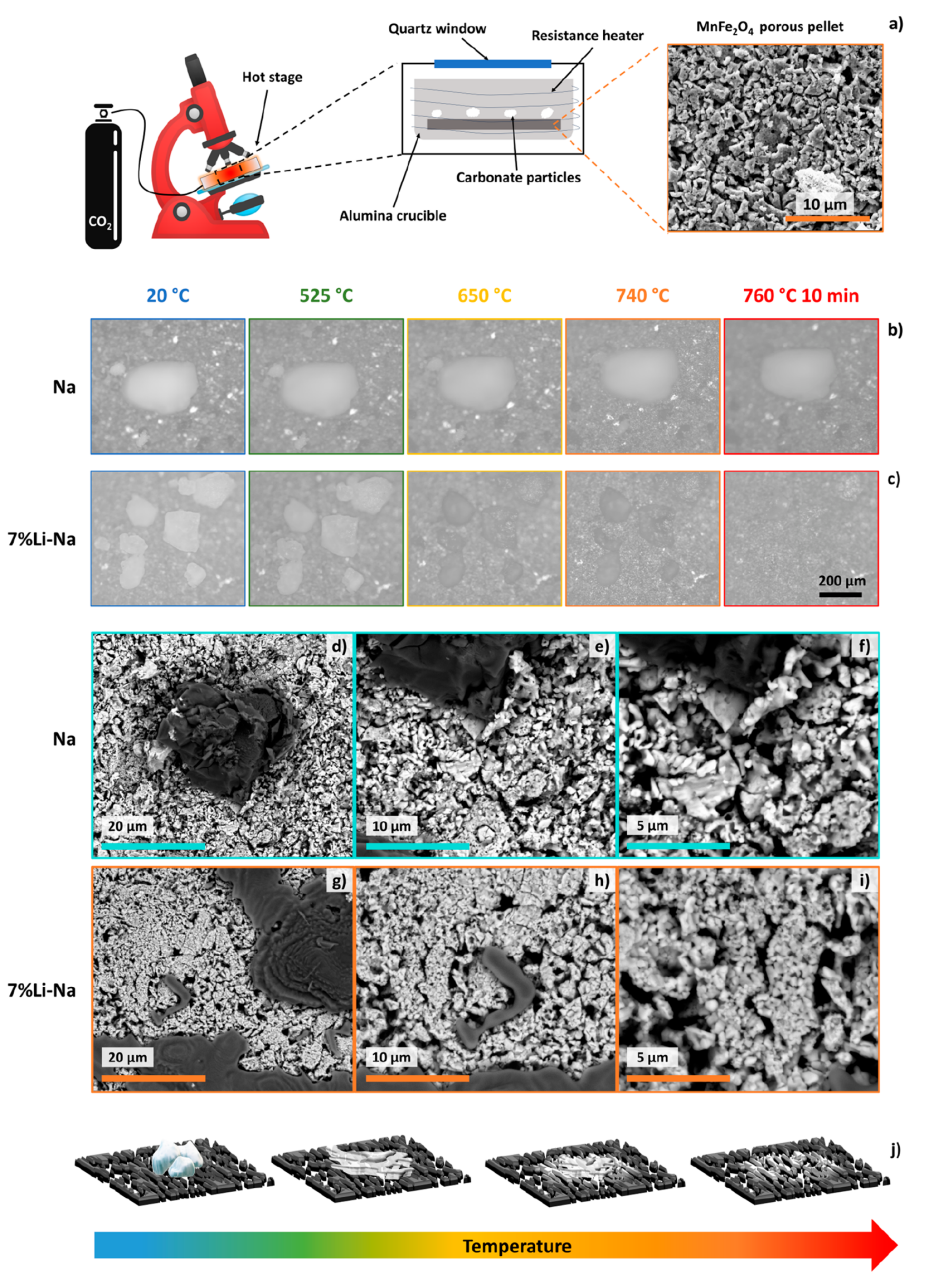
*In situ* high-temperature optical microscopy experiments performed for the Na and 7% Li-Na systems. (a) Scheme of the experimental setup/methodology (full details in section 2.5). *In situ* microstructural evolution of (b) Na_2_CO_3_ and (c) (Na_0.93_Li_0.07_)_2_CO_3_ particles deposited on MnFe_2_O_4_ pellets upon heating from RT to 760 °C under CO_2_. Backscattered SEM micrographs acquired at increasing magnifications after the *in situ* experiments of the (d–f) Na and (g–i) 7% Li-Na samples. (j) Schematic representation of the capillary imbibition of porous MnFe_2_O_4_ during the melting of the (Na_0.93_Li_0.07_)_2_CO_3_ particles upon heating.

The formation of liquid in the 7% Li-Na mixture during the regeneration step at 750 °C under CO_2_ was confirmed by *in situ* optical microscopy heating experiments of porous MnFe_2_O_4_ pellets, on top of which Na_2_CO_3_ and (Na_0.93_Li_0.07_)_2_CO_3_ were deposited (Figure 10). A schematic representation of the overall methodology is reported in Figure 10a, while full experimental details are reported in section 2.5. A completely different microstructural evolution was observed for the Na and the 7% Li-Na mixture, which are reported in Figure 10b, c, respectively. Indeed, while Na_2_CO_3_ particles retained their initial shape even at 760 °C, the eutectoid (Na_0.93_Li_0.07_)_2_CO_3_ particles started vanishing when the MnFe_2_O_4_ pellet surface reached around 650 °C. After 10 min at 760 °C, the powders disappeared completely. As thermogravimetry of (Na_0.93_Li_0.07_)_2_CO_3_ powders ruled out the decomposition under CO_2_ atmosphere even after 1 h at 750 °C (Figure S11), such behavior must be due to the melting of the carbonate. Indeed, the backscattered SEM images of the 7% Li-Na sample after the *in situ* experiment (Figure 10g–i) highlighted that the remaining (Na_0.93_Li_0.07_)_2_CO_3_ that did not have the time to melt showed very smooth and rounded edges (Figure 10d, e); this morphology is typical of molten phases cooled before crystallization could take place.^41^ On the other hand, the Na_2_CO_3_ particles preserved their initial morphology, with sharp and defined edges (Figure 10a–c). As a result, the regions adjacent to the Na_2_CO_3_ and (Na_0.93_Li_0.07_)_2_CO_3_ particles (Figure 10f and 10i, respectively) show quite a different morphology. While the MnFe_2_O_4_ pellet in the Na sample preserved its original porous structure (SEM image on top-right of Figure 10a), the molten (Na_0.93_Li_0.07_)_2_CO_3_ imbibed the MnFe_2_O_4_ pellet underneath and altered its morphology, as schematized in Figure 10j.

### 3.5 Concluding Remarks

Overall, looking at the data in Table 4, it is evident that the reversibility in terms of mass change cannot always be related in a straightforward way to the H_2_ production reversibility. For instance, the thermogravimetry data would suggest a better H_2_ production for the Li-K-Na compared to the 50% Li-Na mixture. However, the H_2_ production data do not point out any significant differences between the two mixtures, as neither showed any reversibility. The same argument can be made for the other mixtures. This is because the contribution of the redox process to the mass change, i.e., O_2_ uptake/release during the water splitting/reduction steps, cannot be distinguished from the contribution of the nonredox process, i.e., CO_2_ release/uptake during the water splitting/reduction steps. Moreover, the mass change due to the O_2_ release/uptake is minor if compared to the mass change related to the decarbonation/carbonation. For instance, the O_2_ uptake during the WS step of the Na mixture corresponds to a 2 wt % mass gain, while the CO_2_ release to a 16.9 wt % mass loss. A more detailed explanation can be found in our previous work.^25^ For the present thermochemical cycle, a good reversibility in terms of mass change can be seen as a *conditio sine qua non* for a reversible H_2_ production. However, a good reversibility in terms of mass change does not automatically imply such reversibility. Thus, calculating the H_2_ efficiency based on thermogravimetric reversibility may lead to errors.^38^

What is certain is that the loss of reversibility in the H_2_ production was due to a decrease in the available redox-active Mn^3+^/Mn^2+^ couple. On the one hand, this can be due to the progressive loss of specific surface area, which is in turn related to sintering and coalescence phenomena. This decreased the alkali cations immediately available to react with CO_2_ during the reduction step to form the corresponding carbonate. After the first carbonate layer is formed, the kinetic of the Na-deintercalation decreased and caused the incomplete regeneration of the Mn^2+^ cations during the reduction step and led to the formation of nonstoichiometric phases.

The preparation of the initial mixture can somehow limit these undesired effects and ensure a decent H_2_ production, even if way below the theoretical yield.^28,29^ Atomic substitution was shown to have a positive effect on H_2_ production reversibility, as shown in our previous work on a Zn-doped Na mixture.^25^ On the other hand, the chemistry of the system played a key role as the introduction of other cations changes the thermodynamics of the system. This could lead to the formation of secondary phases which are highly stable, thus affecting the reversibility of the system. The formation of LiFeO_2_ observed in this work for the 50% Li-Na and Li-K-Na mixture is a striking example.

Moreover, the kinetic and thermodynamic contributions are quite complex to untangle, and this case is not an exception. In particular, the lower melting temperatures of the eutectic/eutectoid mixtures compared to pure Na_2_CO_3_ may also favor the sintering of the powders, thus contributing to the decrease of reactive surfaces and slowing down the regeneration of the reactants. This would explain the faster drop in the H_2_ production observed for 7% Li-Na compared to Na, which is also confirmed by the higher amount of nonstoichiometric Na_*x*_Mn_3_O_7._ Further research will be addressed to evaluate strategies for the improvement of the 7% Li-Na mixture to avoid, or at least limit, the undesired sintering related to the liquid carbonate formation.

## 4 Conclusions

In this work, the effect of pure Na_2_CO_3_ substitution by different eutectic or eutectoid alkali carbonate mixtures in the sodium manganese ferrite thermochemical cycle was investigated. The main aim was to evaluate whether the lower melting point of the eutectic or eutectoid alkali carbonate mixtures affected the MnFe_2_O_4_-carbonate mixture decomposition temperature, the overall cycle reversibility, and the WS reaction kinetics.

The overall cycle reversibility was assessed under nonoxidative and oxidative conditions. Under nonoxidative conditions, the CO_2_ capacity upon cycling indicated that the most reversible mixture was the Na mixture followed closely by the 7% Li-Na mixture. Under oxidative conditions, the Na mixture also exhibited the highest reversibility in terms of hydrogen production followed by the 7% Li-Na mixture. However, all the mixtures suffered from a significant loss of reversibility upon cycling. The XRD and SEM analysis suggest that the loss of reversibility is due to the formation of undesired thermodynamically stable phases and a decrease of reactive surfaces due to sintering. Furthermore, the sintering effect is more pronounced in the MnFe_2_O_4_-mixed carbonate mixtures due to the formation of liquid carbonate during the regeneration under CO_2_ atmosphere.

In contrast, Na_2_CO_3_ substitution by the mixed alkali carbonate mixtures had a positive effect on the WS kinetics. This was particularly evident for the 7% Li-Na mixture. After the first cycle, the 7% Li-Na mixture exhibits a faster kinetics than the Na mixture. Thermodynamic calculations suggested that this enhanced kinetics could be attributed to the fact that the alkali carbonate mixture in the 7% Li-Na is partially in the liquid state when water is introduced after the regeneration step. Such liquid formation was confirmed by *in situ* optical microscopy. All this suggests that lowering the melting point of the carbonate is a suitable strategy to increase the WS kinetics, as the formation of liquid carbonate that wets the metal oxide can help the ion diffusion. Unfortunately, the same liquid favors sintering and leads to a faster loss of reversibility.

We are currently investigating different strategies to decrease the formation of nonstoichiometric phases and to hinder the sintering of the materials and increase the overall reversibility of the cycle.

## Data Availability

The raw/processed data required to reproduce these findings cannot be shared at this time as the data also forms part of an ongoing study.

## Supporting Information Available

Binary phase diagram, thermograms, and DSC profiles for alkali carbonate and MnFe_2_O_4_-alkali carbonate mixtures, melting and decomposition temperatures for alkali carbonate and MnFe_2_O_4_-alkali carbonate mixtures, equilibrium calculations under inert (Ar) and CO_2_ atmosphere, results and disscussion on the reversibility under nonoxidative and oxidative conditions, thermograms during five H_2_ production cycles, H_2_ production and evolution of the H_2_ concentration for the replicates, XRD and SEM analysis of the %7 Li-Na mixture after the first and the second WS reaction step, and temperature profiles using LINKAM hot stage and thermogram profile of (Na_0.93_Li_0.07_)_2_CO_3_ powders under CO_2_ atmosphere (PDF)
