# Supplementary material for: Sodium Manganese Ferrite Water Splitting Cycle: Unravelling the Effect of Solid–Liquid Interfaces in Molten Alkali Carbonates
Source: ACS Appl Mater Interfaces. 2024 Jun 19;16(26):33270–84. doi: 10.1021/acsami.4c00549 (PMC11231967; doi:10.1021/acsami.4c00549)
Supplement: Supplementary file 1 — am4c00549_si_001.pdf [file am4c00549_si_001.pdf]

# Supporting Information

# Sodium manganese ferrite water splitting cycle: unravelling the effect of solid-liquid interfaces in molten alkali carbonates.

Joseba Udaeta[1], Mikel Oregui Bengoechea[1*], Francesco Torre[2**], Nerea Uranga[3], Marta Hernaiz[3], Beatriz Lucio[1], Pedro Luis Arias[1], Elena Palomo del Barrio[2,4], Stefania Doppiu[2].

[1]Department of Chemical and Environmental Engineering, School of Engineering, University of the Basque Country UPV/EHU, Plaza Ingeniero Torres Quevedo, 1. 48013 Bilbao., Spain.

[2]Centre for Cooperative Research on Alternative Energies (CIC energiGUNE), Basque Research and Technology Alliance (BRTA), Alava Technology Park, Albert Einstein 48, 01510 Vitoria-Gasteiz, Spain.

[3]Tekniker, Basque Research and Technology Alliance (BRTA), Parke Teknologikoa, Iñaki Goenaga, 5 20600 Eibar, Gipuzkoa, Spain.

[4]Ikerbasque, Basque Foundation for Science, Bilbao 348013, Spain.

[*]Corresponding authors.

[**]Corresponding authors.

E-mail addresses: mikel.oregui@ehu.eus (M. Oregui), ftorre@cicenergigune.com (F.Torre)

**Keywords:** thermochemical water splitting; sodium manganese ferrite cycle; atomic substitution; carbonation; decarbonation; hydrogen production.

## S1. Phase diagram

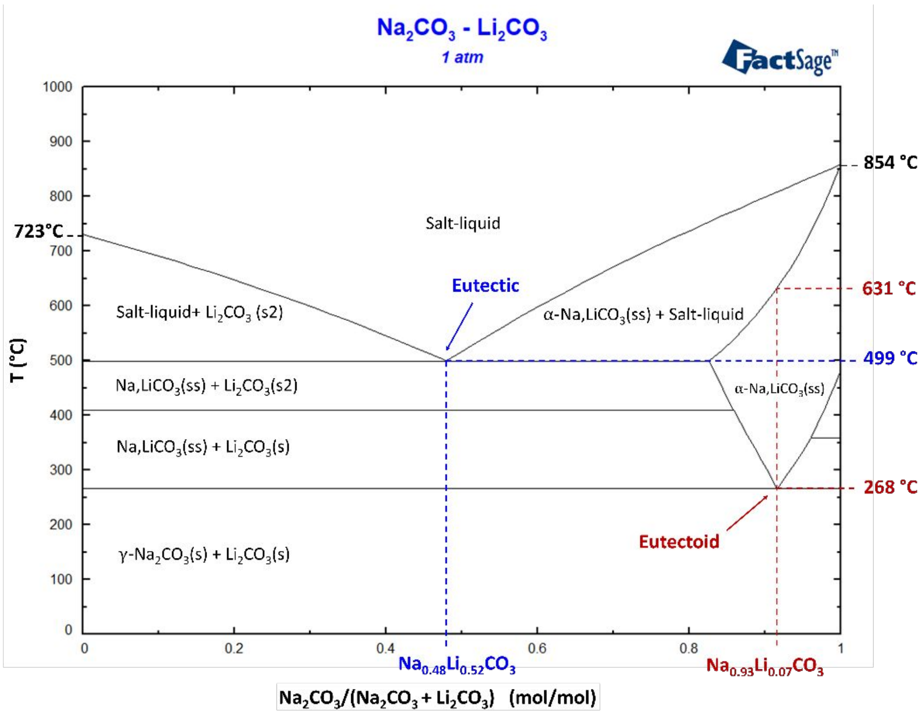


**Figure S1.** Binary phase diagram of the $Na_2CO_3$-$Li_2CO_3$ system. The compositions and transition temperatures of the eutectic ($Na_{0.48}Li_{0.52}CO_3$) and eutectoid ($Na_{0.93}Li_{0.07}CO_3$) mixtures are indicated in blue and red, respectively.

## S2. STA of carbonate and $MnFe_2O_4$-carbonate mixtures

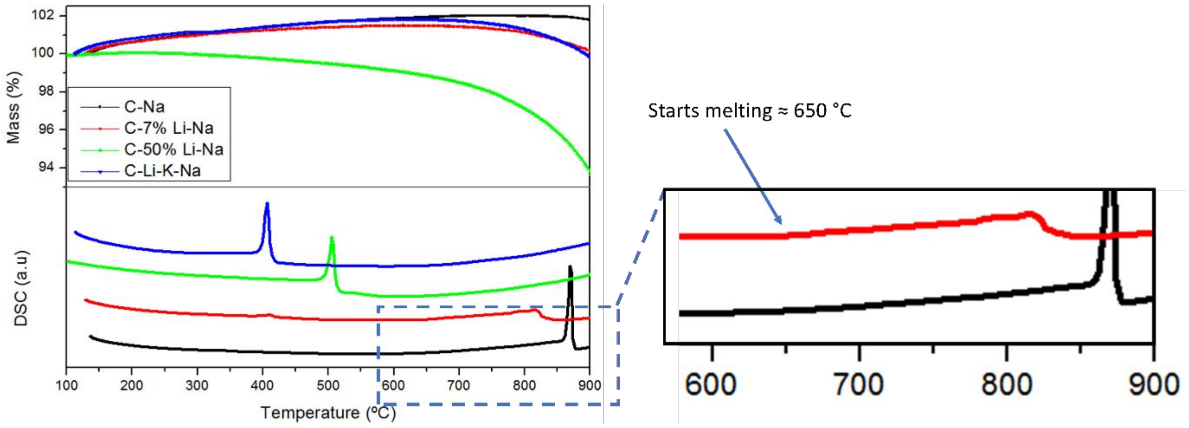


**Figure S2.** Thermograms and DSC profiles for pure $Na_2CO_3$ and the eutectic or eutectoid alkali carbonate mixtures: C-Na (black), C-7%LiNa (red), C-50%Li-Na (green) and C-Li-K-Na (blue).

**Table S1.** Melting and decomposition temperature of pure $Na_2CO_3$ and the eutectic or eutectoid alkali carbonate mixtures

| Mixture | $T_{Melting}$, °C | $T_{Decomposition}$, °C |
|---|---|---|
| **C-Na** | 863 | >900 |
| **C-7%Li-Na** | 650 °C | 874 |
| **C-50%Li-Na** | 498 | 630 |
| **C-Li-K-Na** | 399 | 850 |

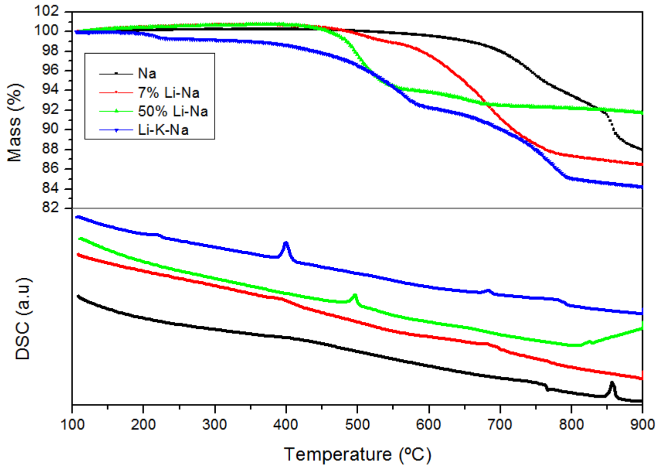


**Figure S3.** Thermograms and DSC profiles for the (1:1) $MnFe_2O_4$-alkali carbonate mixtures: Na (black), 7%LiNa (red), 50%Li-Na (green) and Li-K-Na (blue).

**Table S2.** Decomposition temperature of the $MnFe_2O_4$-alkali carbonate mixtures. The values reported in parentheses are the theoretical values obtained by the equilibrium calculations reported in Figure S4.

| Mixture | $T_{Decomposition}$, °C | | |
|---|---|---|---|
| | **Step 1** | **Step 2** | **Step 3** |
| **Na** | 677 (358) | 850 | - |
| **7%Li-Na** | 470 (279) | 584 (499) | - |
| **50%Li-Na** | 469 (279) | 618 (580) | - |
| **Li-K-Na** | 196 (241) | 478 (599) | 717 (741) |

## S3. Equilibrium calculations under inert atmosphere

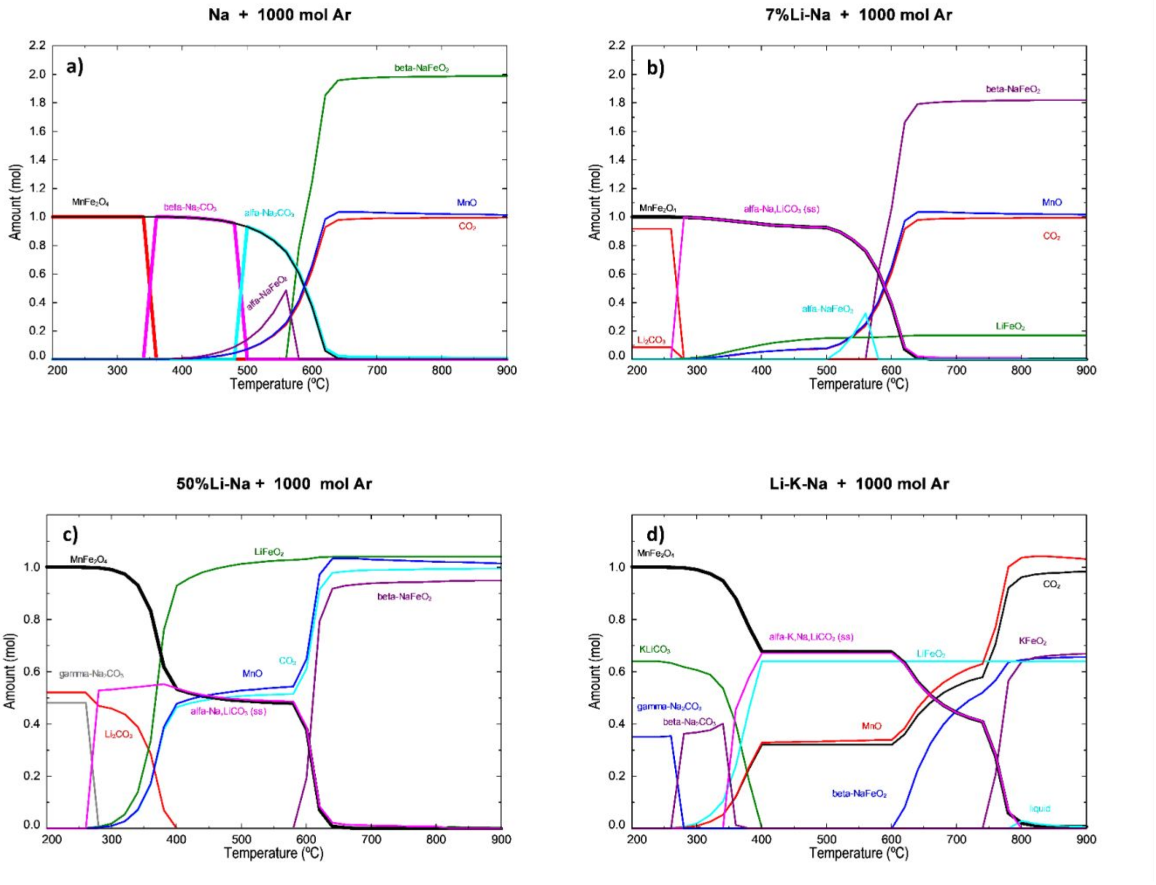


**Figure S4.** Equilibrium calculations of the molar fractions of the different species and phases found at different temperatures under inert atmosphere (Ar) for the Na **(a)**, 7%Li-Na **(b)** and 50%Li-Na **(c)** and Li-K-Na **(d)** mixtures. The calculations were carried out using the FactSage program.

## S4. TG cycling experiments

### S4.1. Reversibility under non-oxidative conditions

The cycled 50%Li-Na showed a significant amount of $LiFeO_2$ (COD 1541312), which explains the lower $CO_2$ capacity of the mixture. In fact, we recently found that the decarbonation of the $MnFe_2O_4$-$Li_2CO_3$ mixture leads to the irreversible intercalation of Li to form $LiFeO_2$[1]. Moreover, the cubic spinel phase regenerated during the final carbonation step is characterized by a lattice constant that is significantly smaller than the one observed for the Na and 7%Li-Na mixtures - i.e., 8.46 vs 8.50 Å. A shrinkage in the crystal lattice of the cubic $MnFe_2O_4$ phase was already observed after cycling a $MnFe_2O_4$-$Li_2CO_3$ mixture and can be attributed to partial Li atomic substitution or intercalation in the $MnFe_2O_4$ lattice.

The XRD of the cycled Li-K-Na mixture indicates $MnFe_2O_4$ as the main phase, followed by traces of $LiFeO_2$, $Na_2CO_3$ and $HK_2Na(CO_3)_2 \cdot H_2O$. This result is somehow in contrast with the low $CO_2$ capacity observed for this mixture. In fact, for the other mixtures, the reversibility loss was always reflected by the presence of significant amounts of $NaFeO_2$ and $LiFeO_2$, i.e. to the incomplete regeneration of the starting reactants. As Li-K-Na shows the lowest reversibility among the four mixtures, higher amounts of these phases would be expected. Moreover, it was previously observed that the $MnFe_2O_4$-$K_2CO_3$ mixture loses $CO_2$ capacity upon cycling due to the formation of potassium beta ferrite ($K_2Fe_{10}O_{16}$)[1]. However, this phase was not detected in the cycled Li-K-Na. Rather, the presence of $HK_2Na(CO_3)_2 \cdot H_2O$ suggests that K tends to form a mixed Na-K carbonate that subsequently absorbed moisture before the XRD analysis was performed. Another interesting point is that the cubic $MnFe_2O_4$ phase in the cycled Li-K-Na presented the same lattice shrinkage observed in the 50%Li-Na, with an average lattice parameter of 8.46 Å. This suggests that part of Li present in the mixture was incorporated in the spinel phase and explains the low amount of $LiFeO_2$.

## S4.2. Hydrogen production cycles under oxidative conditions

During the $H_2$ production experiments, the 50%Li-Na and Li-K-Na exhibited no reversibility in terms of hydrogen production, as they only produced hydrogen in the first cycle. The XRD of both mixtures excludes the formation of appreciable amounts of $Na_xMn_3O_7$ (Figure 6). Rather, the loss of reversibility of 50% Li-Na was clearly due to the formation of $LiFeO_2$, which is in line with the 50% drop in the $CO_2$ capacity observed after the first cycle. Also, K-Li-Na showed the presence of $LiFeO_2$, which explains the loss of $CO_2$ capacity observed by thermogravimetry. These results are in line with the thermodynamic calculations performed under $CO_2$ (Figure S9, Supportin Information). Indeed, at 750 ºC the high amounts of the $LiFeO_2$ phase formed during the WS of the 50 %Li-Na and the Li-K-Na mixtures are predicted to be stable even under $CO_2$.

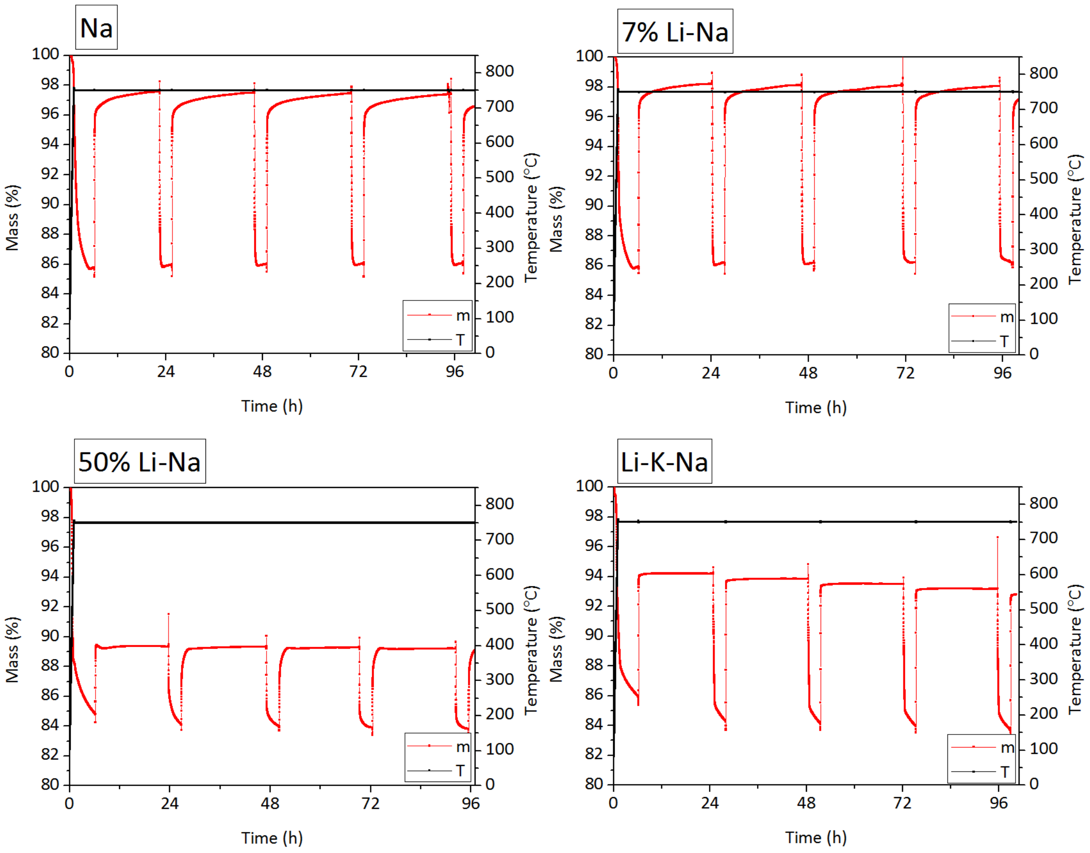


**Figure S5**. Thermograms (red line) obtained for the four mixtures during five $H_2$ production cycles in isothermal conditions at 750 ºC. The temperature measured during the experiments is also reported (black line).

**Table S3.** $H_2$ production for the replicates of the first and second cycle carried out for the 7%Li-Na mixture in order to assess the reproducibility of the hydrogen production measurements.

| Mixture | H₂ production (mmol H₂/g) | |
|---|---|---|
| | Cycle 1 | Cycle 2 |
| **7%Li-Na** | 0.87 | 0.05 |
| | 0.79 | 0.04 |
| | 0.83 | N.A.[a] |
| **Mean** | 0.83±0.04 | 0.045 |

[a]N.A.: not available

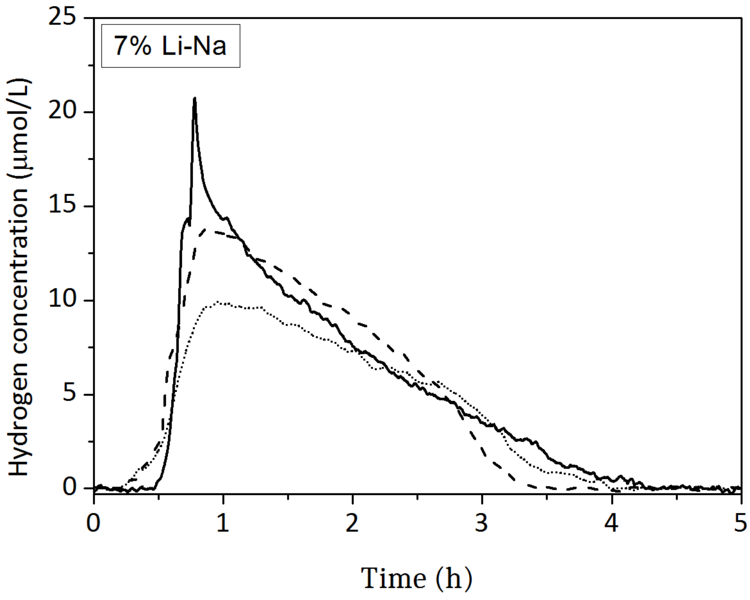


**Figure S6.** The evolution of the $H_2$ hydrogen concentration in the exhaust gases for the replicates of the first cycle carried out for the 7%Li-Na mixture in order to assess the reproducibility of the hydrogen production measurements.

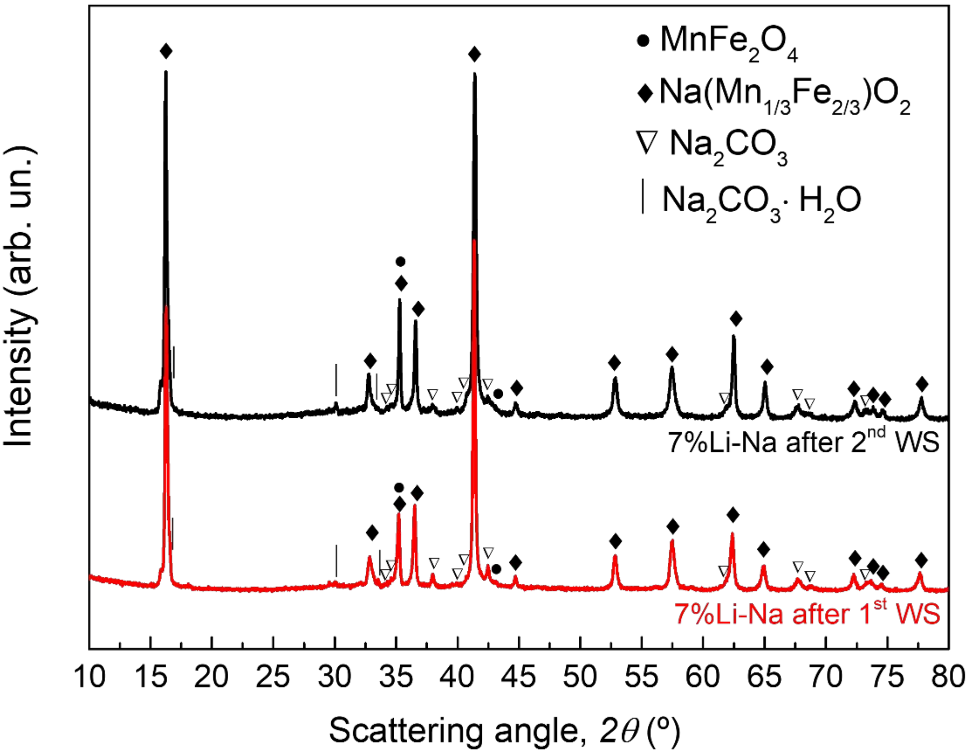


**Figure S7.** XRD analysis of the %7Li-Na mixture after the first and the second WS reaction step.

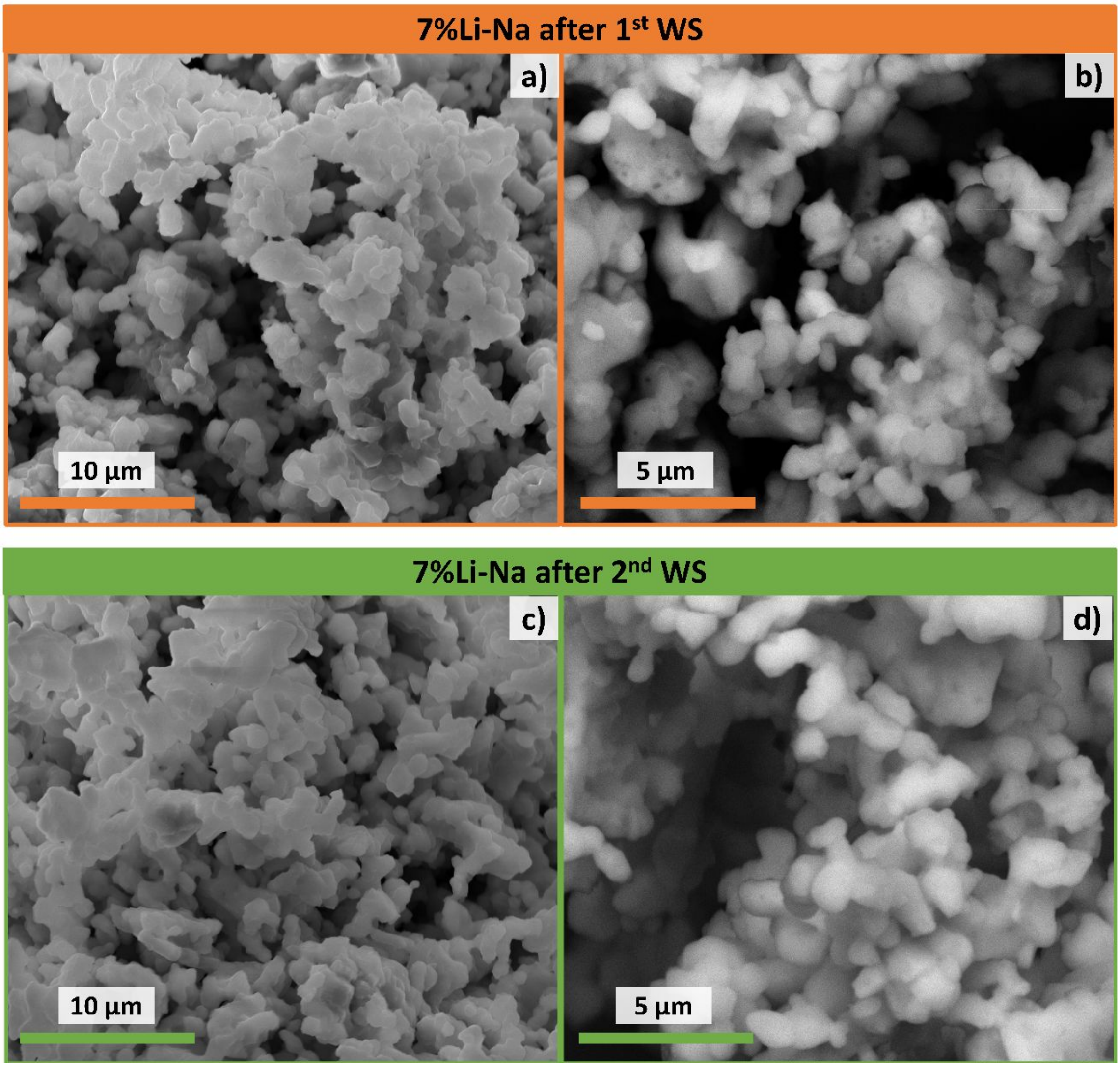


**Figure S8.** SEM analysis of the %7Li-Na mixture after the first (a-b) and the second (c-d) WS reaction step. Secondary electrons (a, c) and (b, d) backscattered electrons images are reported.

## S5. Equilibrium calculations under $CO_2$ atmosphere

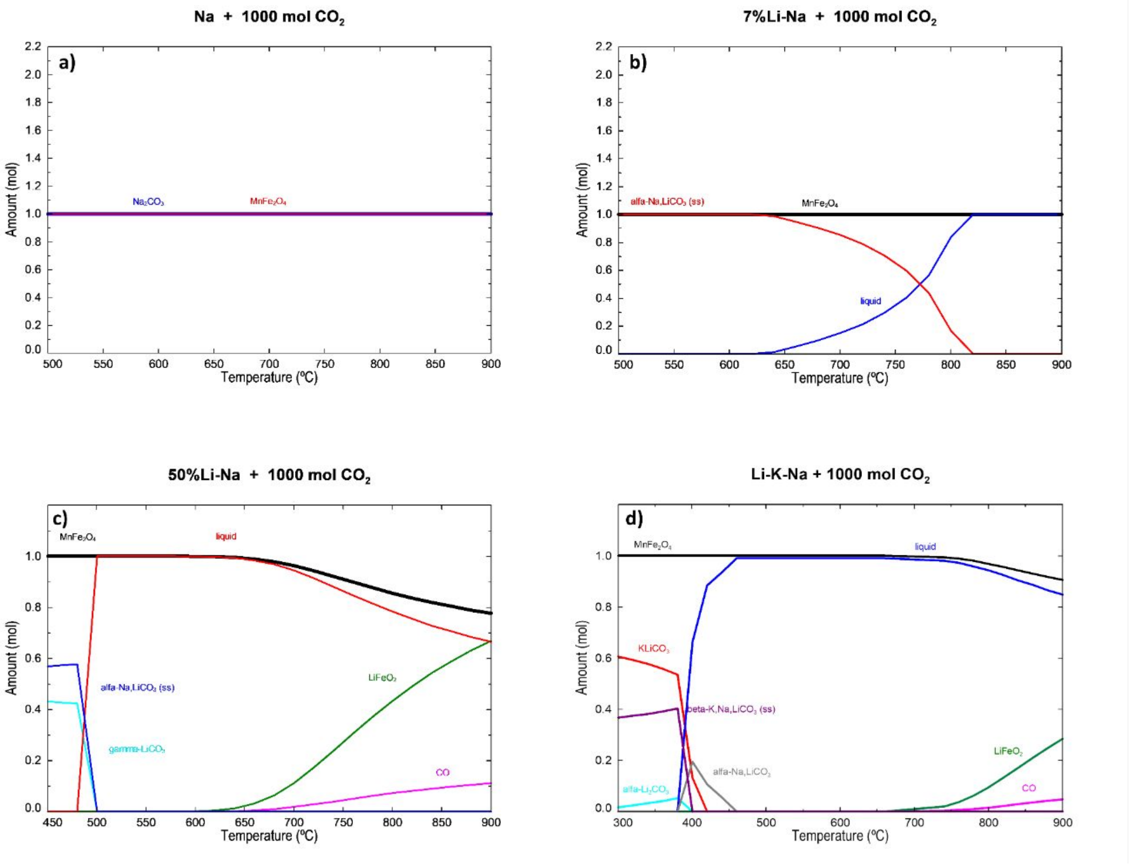


**Figure S9.** Equilibrium calculations of the molar fractions of the different species and phases found at different temperatures under $CO_2$ for the Na **(a)**, 7%Li-Na **(b)** and 50%Li-Na **(c)** and Li-K-Na **(d)** mixtures. The calculations were carried out using the FactSage program.

## S6. Hot-stage optical microscopy

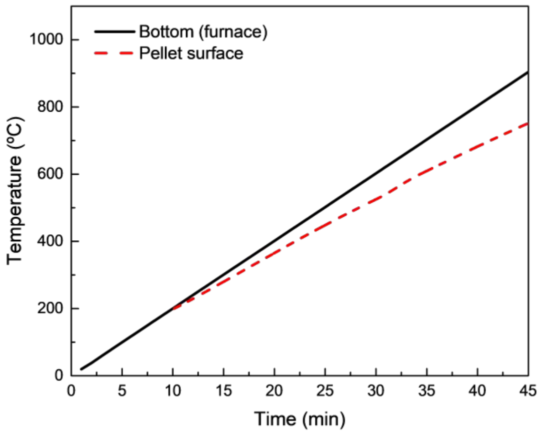


**Figure S10.** Temperature profiles obtained by heating a $MnFe_2O_4$ porous pellet at 20 ºC/min with 50 mL/min of $CO_2$ using the LINKAM hot stage. The black curve refers to the temperature measured by the hot stage thermocouple placed on the bottom of the crucible, while the dashed red curve refers to the surface temperature of the pellet measured by a pyrometer.

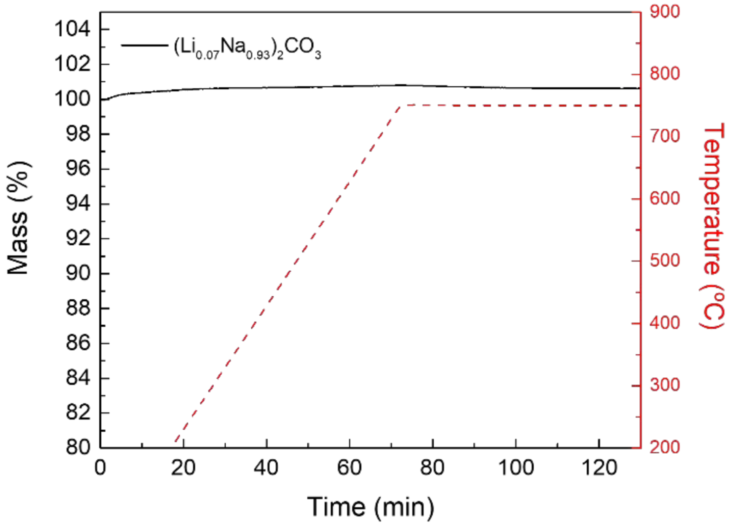


**Figure S11.** Thermogram profile of $(Na_{0.93}Li_{0.07})_2CO_3$ powders under $CO_2$ atmosphere (100 mL/min).

## References

(1) Torre, F.; Sanchez, T. A.; Doppiu, S.; Bengoechea, M. O.; Arias Ergueta, P. L.; Palomo del Barrio, E. Effect of Atomic Substitution on the Sodium Manganese Ferrite Thermochemical Cycle for Hydrogen Production. *Mater Today Energy* **2022**, *29*. https://doi.org/10.1016/j.mtener.2022.101094.
